# Recessive ciliopathy mutations in primary endocardial fibroelastosis: a rare neonatal cardiomyopathy in a case of Alstrom syndrome

**DOI:** 10.1007/s00109-021-02112-z

**Published:** 2021-08-13

**Authors:** Yan Zhao, Lee-kai Wang, Ascia Eskin, Xuedong Kang, Viviana M. Fajardo, Zubin Mehta, Stacy Pineles, Ryan J. Schmidt, Aaron Nagiel, Gary Satou, Meena Garg, Myke Federman, Leigh C. Reardon, Steven L. Lee, Reshma Biniwale, Wayne W. Grody, Nancy Halnon, Negar Khanlou, Fabiola Quintero-Rivera, Juan C. Alejos, Atsushi Nakano, Gregory A. Fishbein, Glen S. Van Arsdell, Stanley F. Nelson, Marlin Touma

**Affiliations:** 1 Department of Pediatrics, 3762 MacDonald Research Laboratories, David Geffen School of Medicine, University of California Los Angeles, 675 Charles E. Young Dr S, CA 90095 Los Angeles, USA; 2 Neonatal/Congenital Heart Laboratory, Cardiovascular Research Laboratory, University of California Los Angeles, Los Angeles, CA USA; 3 Department of Pediatrics, Children’s Discovery and Innovation Institute, David Geffen School of Medicine, University of California Los Angeles, Los Angeles, CA USA; 4 Institute for Precision Health, David Geffen School of Medicine, University of California Los Angeles, Los Angeles, CA USA; 5 Department of Human Genetics, David Geffen School of Medicine, University of California Los Angeles, Los Angeles, CA USA; 6 Department of Ophthalmology, David Geffen School of Medicine, University of California Los Angeles, Los Angeles, CA USA; 7 Department of Pathology and Laboratory Medicine, Children’s Hospital Los Angeles, Los Angeles, CA USA; 8 The Vision Center, Department of Surgery, Children’s Hospital Los Angeles, Los Angeles, CA USA; 9 Department of Ophthalmology, Roski Eye Institute, University of Southern California, Los Angeles, CA USA; 10 Ahmanson/UCLA Adult Congenital Heart Disease Center, Department of Medicine, David Geffen School of Medicine, University of California Los Angeles, Los Angeles, CA USA; 11 Department of Cardiothoracic Surgery, David Geffen School of Medicine, University of California Los Angeles, Los Angeles, CA USA; 12 Department of Pathology and Laboratory Medicine, David Geffen School of Medicine, University of California Los Angeles, Los Angeles, CA USA; 13 Department of Pathology and Laboratory Medicine and Department of Pediatrics, University of California Irvine, CA Irvine, USA; 14 Eli and Edythe Broad Stem Cell Research Center, David Geffen School of Medicine, University of California Los Angeles, Los Angeles, CA USA; 15 The Molecular Biology Institute, David Geffen School of Medicine, University of California Los Angeles, Los Angeles, CA USA

**Keywords:** Primary endocardial fibroelastosis, Neonatal cardiomyopathy, Alstrom syndrome, Exome sequencing, RNA sequencing, Fibrosis, Epithelial mesenchymal transition, Retinal dystrophy, Rare undiagnosed disease

## Abstract

**Abstract:**

Among neonatal cardiomyopathies, primary endocardial fibroelastosis (pEFE) remains a mysterious disease of the endomyocardium that is poorly genetically characterized, affecting 1/5000 live births and accounting for 25% of the entire pediatric dilated cardiomyopathy (DCM) with a devastating course and grave prognosis. To investigate the potential genetic contribution to pEFE, we performed integrative genomic analysis, using whole exome sequencing (WES) and RNA-seq in a female infant with confirmed pathological diagnosis of pEFE. Within regions of homozygosity in the proband genome, WES analysis revealed novel parent-transmitted homozygous mutations affecting three genes with known roles in cilia assembly or function. Among them, a novel homozygous variant [c.1943delA] of uncertain significance in *ALMS1* was prioritized for functional genomic and mechanistic analysis. Loss of function mutations of *ALMS1* have been implicated in Alstrom syndrome (AS) [OMIM 203800], a rare recessive ciliopathy that has been associated with cardiomyopathy. The variant of interest results in a frameshift introducing a premature stop codon. RNA-seq of the proband’s dermal fibroblasts confirmed the impact of the novel *ALMS1* variant on RNA-seq reads and revealed dysregulated cellular signaling and function, including the induction of epithelial mesenchymal transition (EMT) and activation of TGFβ signaling. ALMS1 loss enhanced cellular migration in patient fibroblasts as well as neonatal cardiac fibroblasts, while ALMS1-depleted cardiomyocytes exhibited enhanced proliferation activity. Herein, we present the unique pathological features of pEFE compared to DCM and utilize integrated genomic analysis to elucidate the molecular impact of a novel mutation in *ALMS1* gene in an AS case. Our report provides insights into pEFE etiology and suggests, for the first time to our knowledge, ciliopathy as a potential underlying mechanism for this poorly understood and incurable form of neonatal cardiomyopathy.

**Key message:**

- Primary endocardial fibroelastosis (pEFE) is a rare form of neonatal cardiomyopathy that occurs in 1/5000 live births with significant consequences but unknown etiology.
- Integrated genomics analysis (whole exome sequencing and RNA sequencing) elucidates novel genetic contribution to pEFE etiology.
- In this case, the cardiac manifestation in Alstrom syndrome is pEFE. To our knowledge, this report provides the first evidence linking ciliopathy to pEFE etiology.
- Infants with pEFE should be examined for syndromic features of Alstrom syndrome.
- Our findings lead to a better understanding of the molecular mechanisms of pEFE, paving the way to potential diagnostic and therapeutic applications.

**Supplementary information:**

The online version contains supplementary material available at 10.1007/s00109-021-02112-z.

## Background

Cardiomyopathy is a heterogenous group of disorders mainly characterized by abnormal ventricular hypertrophy or dilation leading to ventricular dysfunction [1, 2]. Inherited forms of cardiomyopathy can be broadly classified into hypertrophic cardiomyopathy (HCM), which represents the most commonly inherited form, dilated cardiomyopathy (DCM), restrictive cardiomyopathy (RSM), and arrhythmogenic cardiomyopathy (ACM) [1]. Mendelian inheritance appears to exist in approximately 50% of pediatric DCM. As in other cardiomyopathy forms, mitochondrial disorders, metabolic disease, chromosomal defects, and dysmorphic syndromes may underlie the majority of cases that present early during infancy. Monogenic autosomal and X-linked inheritance have also been reported in DCM cases that present in children less than 18 years of age, of which a substantial subgroup of DCM cases has been associated with skeletal myopathy and/or conduction system disease. At present however, 43% of pediatric DCM cases remain with unidentified cause. Elucidating the underlying genetic etiology is crucial to provide early diagnosis, risk stratification, prognostication, and follow-up of at risk relatives.

Neonatal cardiomyopathies are multifactorial disorders that manifest early after birth as an isolated trait or as a part of a syndromic picture [3]. Syndromic cardiomyopathies are characterized by severe, early-onset ventricular dysfunction accompanied by multisystem disorders. Remarkably, the features of different syndromes may overlap [3, 4]. Hence, the complexity and rarity of these disorders, together with delayed systemic manifestations, different expressivity, and early lethality, make accurate and timely diagnosis difficult. Although we do not fully understand the underlying etiology of these rare and serious disorders, genetic defects play vital roles and may converge on common molecular pathways to different degrees. Next-generation sequencing of DNA and RNA has proved efficient for the early detection of cardiomyopathies allowing the discovery of many genes associated with isolated or syndromic forms, paving the way for elucidating the disease mechanism and identifying disease-specific therapy [5, 6].

Primary endocardial fibroelastosis (pEFE) is a rare form of neonatal DCM that occurs in 1/5000 live births with significant consequences but unknown etiology [7–11]. It is one of the most challenging diseases of the endomyocardium, in which the deposition of sub-endocardial fibrous tissue during early postnatal heart development leads to significant thickening of the endocardium and restriction of left ventricular filling and growth [7]. Patients with pEFE present during early infancy with progressive left ventricular (LV) dysfunction leading to congestive heart failure and early death in 80% of cases. A published report from the UCLA-pediatric heart transplant program indicates that pEFE accounts for approximately 25% of the entire pediatric DCM cohort, while comprising 80% of cardiac transplantation undertaken for patients with DCM before 1 year of age [7]. In contrast to secondary EFE that occurs in association with structural heart malformation such as hypoplastic left ventricle, the anatomy of pEFE hearts is generally normal [7]. Compared to other DCM subtypes, the phenotypic features of pEFE are unique and consistent [7]. The LV is usually dilated with extensive thickening of the endocardium by diffuse elastin and collagen fibers that limit ventricular diastolic compliance. The thickening further extends to the mitral valve and chordae tendineae leading to upward displacement of the papillary muscles and mitral regurgitation. Histologically, sub-endocardial vacuolization of the myocytes with perinuclear clearing has been described [7]. At the ultrastructural level, myofibrillar disarray, widening Z-bands, disorganized intercalated disks, myofibrils’ loss, perinuclear glycogen deposits, and variation in mitochondrial morphology were observed in electron micrographs [11]. Despite being recognized as a distinct pathological entity, the diagnosis of pEFE is entirely based on post-mortem or post-transplant, pathological examination of the diseased heart.

The genetic model of pEFE remains unresolved. Familial aggregation of pEFE has been reported ranging between 8 and 18%; however, remarkable genetic heterogeneity exists [12, 13]. Mutations of the *TAZ* gene, which encodes a mitochondrial targeted acyltransferase known as tafazzin, have been implicated in an X-linked form of pEFE in association with features of BARTH syndrome [OMIM 300394] [13]. However, the genetic mechanism and the mode of inheritance remain unknown in the vast majority of pEFE cases where sporadic isolated cardiomyopathy is presented with negative family history. Since cardiac transplantation is becoming more feasible for infants with end-stage heart failure, establishing an early diagnosis is essential to clinicians in making decisions regarding transplant candidacy. Moreover, elucidating the disease mechanism is necessary to identify targeted therapies. However, this task has been challenging due to the multifaceted complexity of myocardial disease in general, and particularly due to the critical gaps in our knowledge of this disease entity and the limited success in disease modeling necessary for in-depth mechanistic interrogation.

Aiming to elucidate the genetic contribution to pEFE, we carried out an integrative genomic analysis, using trio whole exome sequencing (WES) and RNA-seq, on a female proband with confirmed diagnosis of pEFE and her unaffected parents. We identified a novel homozygous single nucleotide deletion, which results in frameshift [p.val649fs] and introduction of a premature stop codon in *ALMS1*, suggesting the genetic diagnosis of Alstrom syndrome (AS) [OMIM: 203800], an extremely rare, autosomal recessive, Mendelian disorder with a prevalence of less than 1 per million in the general population [14–22]. As a ciliopathy disorder, AS typically manifests during infancy or childhood, although some features may occur later in life, typically associating with severe multi-organ defects. Patients develop progressive cone-rod dystrophy leading to blindness, neuronal hearing loss, early-onset truncal obesity, hypertriglyceridemia, insulin resistance, type 2 diabetes, and short stature, among other features. Patients are also susceptible to multi-organ failure secondary to widespread fibrosis of unknown etiology. Reportedly, cardiomyopathy is an important feature of AS and may manifest during infancy, as a mitogenic cardiomyopathy [18–20], or later in childhood as dilated cardiomyopathy [21, 22]. However, the pEFE phenotype was never described in association with AS. Herein, close clinical follow-up and integrative genomic analysis of WES and RNA-seq of the proband-derived fibroblasts confirmed the causality of the novel *ALMS1* variant and elucidated the molecular signature of pEFE learned from our index AS case. It has been well established that cilia play an important role in cardiac development and congenital heart disease [23]. To our knowledge, our report provides the first evidence linking AS ciliopathy to pEFE etiology.

## Methods

Please refer to Supplemental Materials for detailed methods.

### Human studies

All human studies were conducted in accordance with regulation of the University of California Los Angeles (UCLA) Institutional Review Board (IRB). Subjects provided written informed consent to participate in this study. Electronic medical record, family pedigree, and specimen collection were acquired through the UCLA Congenital Heart Defect (CHD) BioCore [24] following the UCLA-IRB-approved protocols. Specimens were de-identified and coded following acquisition. pEFE diagnosis was determined based on clinical pathological examination of the explanted heart.

### Statistics

Quantified results are presented as mean ± SEM. Student’s *t* test (unpaired, 2-tailed) and ANOVA with post hoc Kruskal–Wallis were used for comparing 2 groups and more than 2 groups, respectively; *P* value less than or equal to 0.05 was considered significant, unless specified otherwise. The correlation of gene expression for each mRNA/trait pair was calculated using Pearson’s correlation and Benjamini–Hochberg correction methods. A Benjamini-Hochberg-adjusted correlation *P* value less than or equal to 0.05 was considered significant.

## Results

### Human studies

All human studies were conducted in accordance with regulation of the University of California Los Angeles (UCLA) Institutional Review Board (IRB). Subjects provided written informed consent to participate in this study. Electronic medical record, family pedigree, and specimen collection were acquired through the UCLA Congenital Heart Defect (CHD) BioCore [24] following the UCLA-IRB-approved protocols. Specimens were de-identified and coded following acquisition.

### Case of interest

**Fig. 1:**
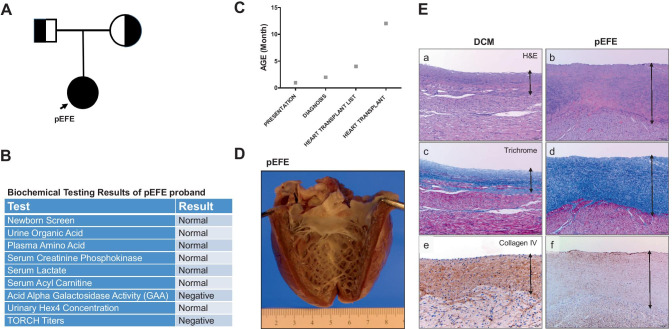
Primary endocardial fibroelastosis (pEFE) is a distinct pathological entity. (**A**) Family pedigree of the pEFE proband affected with novel bi-allelic recessive mutations in three ciliary genes, *DNAH8*, *DNAH17*, and *ALMS1*. (**B**) Table summary of clinical biochemical testing results for the pEFE proband. (**C**) Clinical time course for the pEFE patient. Age at presentation, age at diagnosis, age at listing for heart transplantation, and age at heart transplantation are presented. (**D**) Gross pathology image of pEFE heart demonstrates thickened endocardium. (**E**) Microscopy images of LV sections from a DCM heart (left panel) and a pEFE heart (right panel) demonstrate significant endocardial thickening contributed by deposition of elastic fibrous tissues, while collagen IV was less prominent in pEFE compared to DCM. (**a**, **b**) H&E histochemistry; (**c**, **d**) trichrome histochemistry; (**e**, **f**) collagen IV immunohistochemistry (IHC)

The pEFE proband is a female infant of healthy parents of Hispanic descent, who presented with profound congestive heart failure at 6 weeks of life. The prenatal course and delivery history were uneventful. Newborn screening, infectious work-up, and comprehensive metabolic panel revealed no abnormalities (Fig. 1A, B). pEFE diagnosis was determined based on clinical pathological examination of the explanted heart. No structural defect was identified. At the time of heart transplantation, the ejection fraction EF of the left ventricle was less than 20%. The clinical time course of the participant is summarized in Fig. 1C.

### Pathological findings

Pathological examination was performed by an expert anatomic pathologist. A gross image of the explanted pEFE heart is shown in Fig. 1D. Diffuse thickening of the LV endocardium with an elevation of the papillary muscles and thickening of the free edges of mitral valve leaflets were observed, giving an opaque appearance to the endocardial surface. Compared to an age-matched heart with DCM, the LV endocardial thickness is significantly greater in pEFE (Fig. 1E (a, b)) with dense elastic fibers arranged in a longitudinal pattern shown by trichrome staining (Fig. 1E (c, d)). In contrast, collagen IV deposits were less pronounced (Fig. 1E (e, f)). Based on these pathological features, a clear distinction could be made between pEFE heart and DCM heart.

### Potential contribution of recessive ciliopathy genes to pEFE etiology

**Fig. 2:**
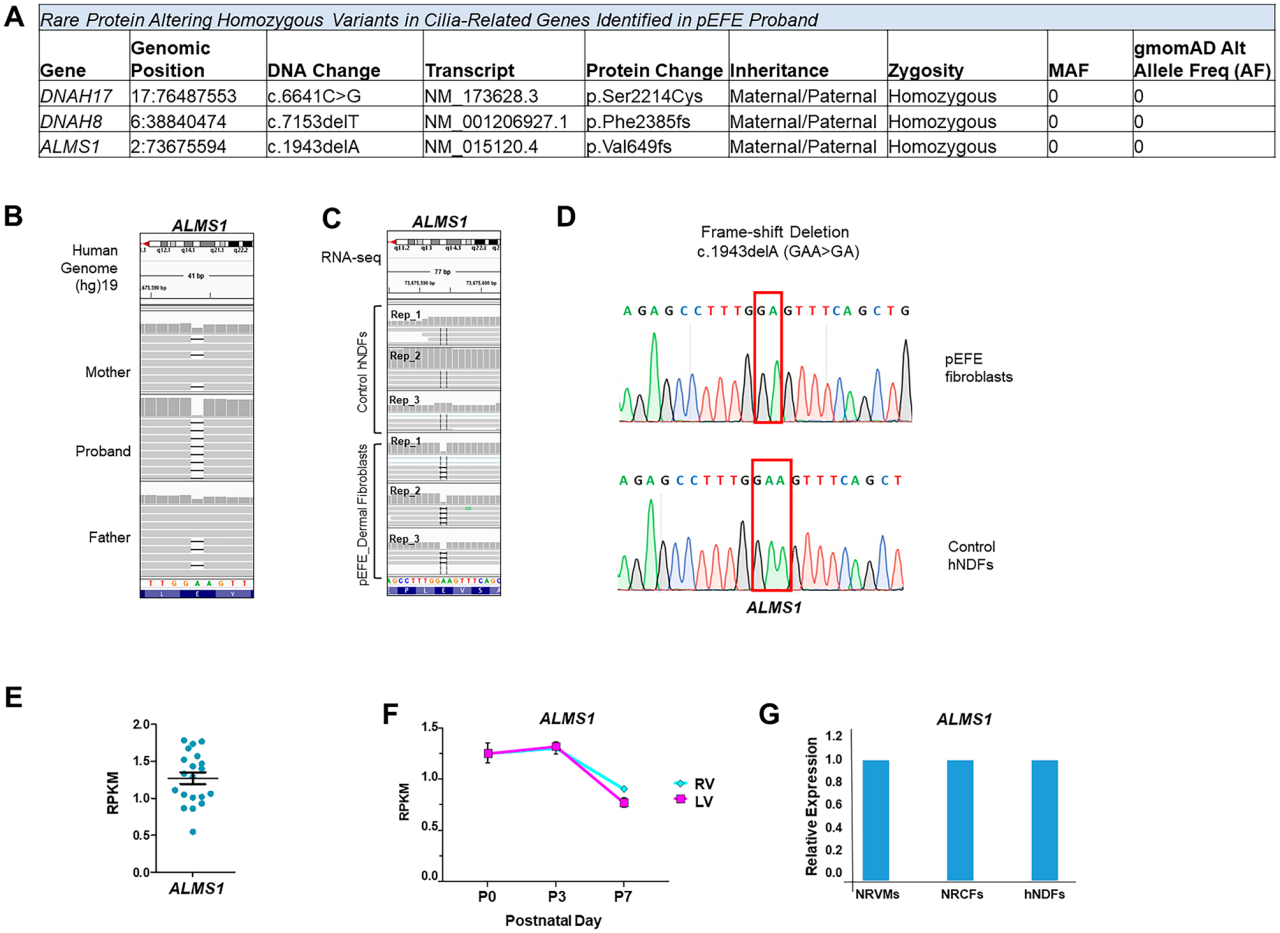
Integrated genomic (WES/RNA-seq) analysis for variant prioritization in pEFE. (**A**) Table summary of the deleterious bi-allelic variants in cilia-related genes detected in the pEFE proband. (**B**) Integrative Genomics Viewer window of Trio WES for the pEFE family shows homozygous deletions in *ALMS1*. The parents are heterozygous carriers for the variant. (**C**) IGV window of RNA-seq demonstrates the consequence of *ALMS1* variant on *ALMS1* RNA reads in pEFE proband-derived fibroblasts compared with control human neonatal dermal fibroblasts (hNDFs). *N* = 3 biological replicates per group. (**D**) Sanger sequencing confirms the bi-allelic *ALMS1* variant in pEFE patient-derived dermal fibroblasts, but not in control hNDFs. (**E**) RNA-seq-derived expression values of *ALMS1* in human congenital heart defect specimens. RPKM: Reads per kilo base per million of mapping reads. (**F**) RNA-seq-derived expression time course of *ALMS1* in neonatal mouse heart right ventricle (RV) and left ventricle (LV) at postnatal day 0 (P0), P3, and P7. (**G**) Expression analysis of *ALMS1* in neonatal rat ventricular myocytes (NRVMs), neonatal rat cardiac fibroblasts (NRCFs), and hNDFs (qRT-PCR). Only *ALMS1* was expressed in all three cell types

 To investigate the genetic contribution to pEFE etiology, we performed WES on our pEFE proband and her parents combined with RNA-seq analysis of pEFE proband dermal fibroblasts (Supplemental Fig. 1). Parents received pre-test genetic counseling and were then asked to provide informed consent to make anonymous clinical and genomic data available for research and publication. Using a custom-made primary gene list of 44 known cardiomyopathy genes, including *TAZ* (Supplemental Table 1), no pathogenic variants were identified. Based on the sporadic nature of pEFE presentation in the proband cohort, we expected that de novo germline mutations might contribute to the genetic basis of pEFE. However, no de novo mutation was detected. The family pedigree did not reveal parental consanguinity or another affected family member. On the contrary, homozygosity analysis revealed 8 large runs of homozygosity (ROH) greater than 5 MBs, totaling 51.8 MBs, which correspond to 1.62% of the proband’s genome (Supplemental Table 2), suggesting that both parents descend from a common, genetically isolated community. Importantly, within these ROHs, three novel homozygous variants in cilia-related genes, *DNAH8*, *DNAH17*, and *ALMS1*, were detected, while both of the unaffected parents were heterozygous carriers for each of these variants (Fig. 2A). The detected variants have not been previously reported in gnomAD, ClinVar (https://www.ncbi.nlm.nih.gov/clinvar/), the 1000 Genomes database (http://www.internationalgenome.org), or ExAC (http://exac.broadinstitute.org/).

*DNAH8* and *DNAH17* genes encode two heavy chain proteins of the axonemal dynein that generate force through ATP hydrolyzing and microtubule binding. The novel homozygous variant in *DNAH17* [17:76,487,553 c.6641C > G] replaces a serine residue for cysteine at the amino acid 2214 (p.Ser2214Cys) and is predicted to be deleterious. The novel homozygous single nucleotide deletion in *DNAH8* [6:38,840,474 c.7153delT] results in a frame shift [p.Phe2385fs] that causes a premature termination of translation leading to a truncated protein. However, *DNAH17* and *DNAH8* mutations have been clinically implicated in spermatogenic failure [OMIM: 618643] [25] and have never been reported in association with cardiomyopathy. Therefore, the novel variants detected in these two genes are unlikely to contribute to pEFE phenotype.

*ALMS1* encodes the centrosome and basal body-associated protein 1, which functions in the formation and maintenance of cilia and in microtubule organization [14, 15]. The novel *ALMS1* variant [2: 73,675,594 c.1943delA] is a single nucleotide deletion, which results in a frameshift [p.Val649fs] and introduction of a premature stop codon. Mutations in *ALMS1* are known causal of AS [OMIM 203800] [14–17], a rare autosomal recessive ciliopathy, which has been associated with cardiomyopathy [16–20], suggesting *ALMS1* as the potential candidate causal gene in our proband. However, since AS has not been associated with pEFE phenotype and our patient presented initially with isolated cardiomyopathy with no other features of AS, further molecular studies are essential to confirm the causality.

### Characterizing the novel ALMS1 variant for functional assessment

Integrative Genomics Viewer (IGV) analysis confirmed the zygosity of the variants (Fig. 2B).

To ascertain that *ALMS1* is the candidate causal gene, we set out first to confirm the impact of these variants on RNA expression. We isolated total RNAs from pEFE proband dermal fibroblasts derived from a punch skin biopsy of the pEFE proband and from hNDFs as a control, and performed paired-end RNA-seq as we previously described [26, 27]. *ALMS1* was expressed in dermal fibroblasts with good read coverage. IGV confirmed the impact of the novel *ALMS1* c.1943delA variant at the RNA level in pEFE fibroblast compared to control hNDF (Fig. 2C). By Sanger sequencing, we confirmed the newly identified homozygous variant in *ALMS1* using gDNA obtained from dermal fibroblasts of the pEFE proband, with human neonatal dermal fibroblasts (hNDFs) used as a negative control (Fig. 2D). Next, we confirmed *ALMS1* expression in heart tissue samples using our previously reported RNA-seq data of congenital heart defects samples obtained from infants with structural heart defects [24, 26] (Fig. 2E).

Given that the cardiac dysfunction of pEFE manifests clinically during the early postnatal period, we next examined *ALMS1* expression in our previously reported RNA-seq data derived from neonatal mouse heart left and right ventricles at postnatal day P0, P3, and P7 [27], available in the NCBI’s Gene Expression Omnibus repository under the Neonatal Heart Maturation SuperSeries (http://www.ncbi.nlm.nih.gov/geo/query/acc.cgi?acc=GSE85728). Again, we demonstrated that *ALMS1* is expressed in both ventricular chambers, exhibiting dynamic regulation in neonatal heart during perinatal stages (Fig. 2F). To further ascertain the expression pattern of *ALMS1* in neonatal heart at the cellular level, we examined its expression in primary cultured neonatal rat ventricular myocytes (NRVMs) and neonatal rat cardiac fibroblasts (NRCFs) isolated from neonatal rat hearts. Indeed, *ALMS1* was expressed in both cardiac cell types (Fig. 2G). Altogether, our findings indicate that *ALMS1*, which is expressed in neonatal heart, has been associated with cardiomyopathy, as the candidate causal gene in pEFE phenotype. Therefore, we focused the following studies to determine the molecular and cellular impact of the novel *ALMS1* variant in pEFE.

### ALMS1 protein expression is absent in pEFE proband dermal fibroblasts *ALMS1*

**Fig. 3:**
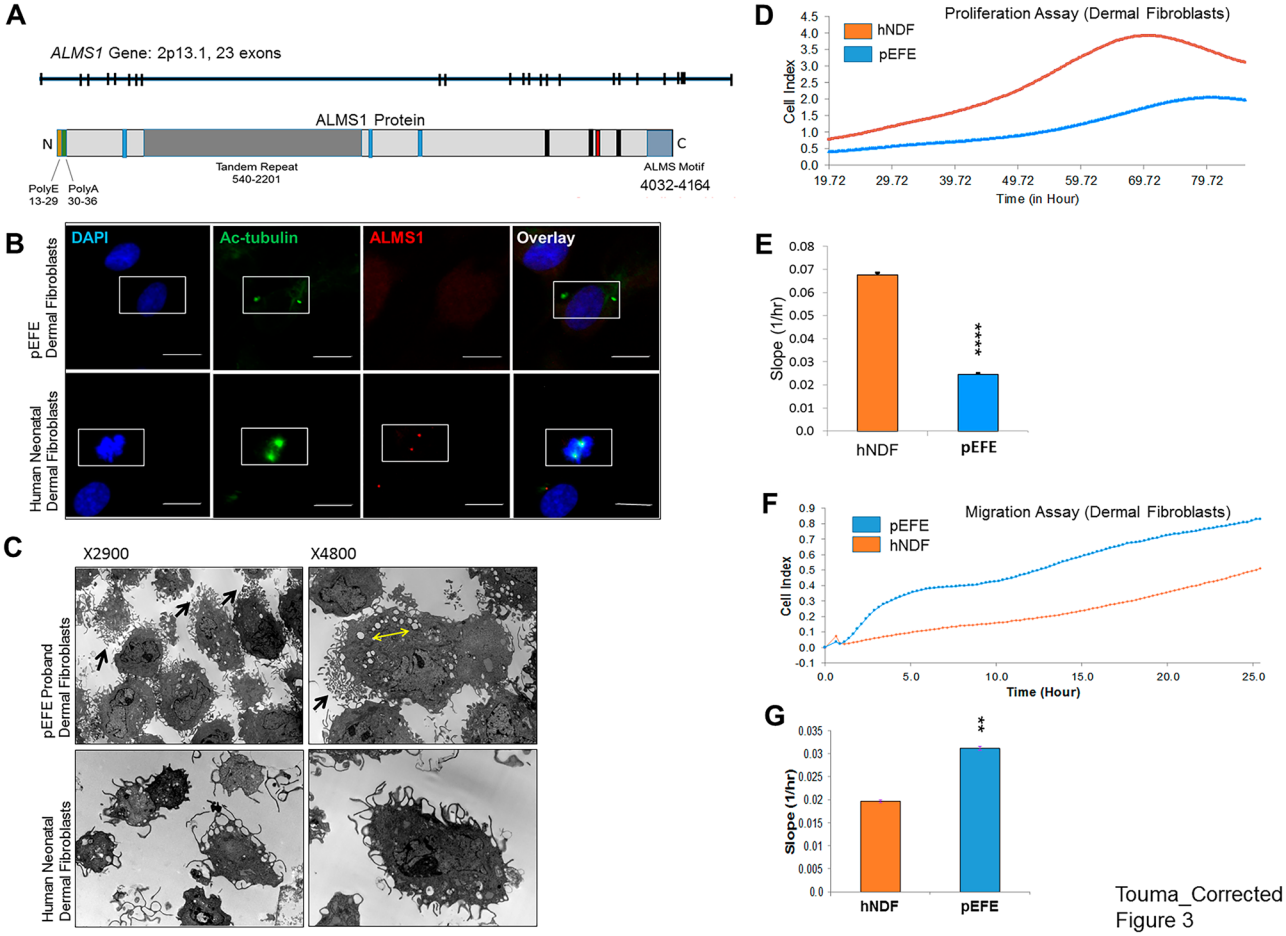
The novel *ALMS1* variant impedes ALMS1 protein expression and alters the functional phenotype of pEFE fibroblasts. (**A**) Schematic representation of ALMS1 gene (upper) and protein with known structural motifs (lower). (**B**) Representative fluorescence microscopy images of ALMS1 immunocytochemistry (ICC) in pEFE-derived fibroblast (upper panel) and hNDF (lower panel). (**C**) Representative electron microscopy (EM) images of pEFE proband and hNDF fibroblasts show unipolar localization of thin branched microvilli was observed in pEFE proband fibroblasts compared to hNDFs (black arrows). Increased intracellular vacuolization was also observed (yellow double-headed arrow). (**D**) Proliferation assay of pEFE dermal fibroblasts and control hNDFs. pEFE proband fibroblasts exhibited lower proliferation activity as demonstrated by lower cellular index over time. The proliferation assay was performed using an xCELLigence RTCA SP instrument over an 85-h period. 2500 cells per well were seeded into the 96-well RTCA E-plate. *N* = 6 biological replicates per group. (**E**) Quantitative analysis of fibroblast proliferation assay shown in (**D**). (**F**) Migration assay of pEFE proband fibroblasts and control hNDFs. pEFE proband fibroblasts exhibited enhanced migration activity as demonstrated by higher cellular index over time. The migration assay was performed using an xCELLigence RTCA DP instrument over a 25-h period. 10,000 cells per well were seeded into the upper chamber of CIM-Plate16 . *N* = 3 biological replicates per group. (**G**) Quantitative analysis of fibroblast migration assay shown in (**F**)

 gene is located on chromosome 2p13.1 spanning 23 exons. It encodes a protein of 4,169 amino acids (461.2 kDa), which lacks known catalytic domains [28, 29], but has several sequence features of unknown function, including a large tandem repeat domain (TRD), three short coiled-coil domains, and a stretch of ~ 130 residues at the C-terminus termed as the ALMS motif [aa: 4032–4164 (Ensembl)] (Fig. 3A). The ALMS1 protein is widely expressed and co*-*localizes with the centrosomes and basal bodies of ciliated cells in different tissues and organs, where ALMS1 has been shown to play important roles in ciliary function and intracellular trafficking [28]. Deletion analysis suggests that the ALMS motif may play a key role in centrosome-targeting [29]. Importantly, the variant of interest [c.1943delA] is predicted to result in a severely truncated ALMS1 protein [p.Val649fs], missing most of the tandem repeats and the highly conserved ALMS motif at the C-terminus. Based on these previously reported observations, we predicted the ALMS1 protein expression and/or stability in the centrioles to be affected by the novel *ALMS1* variant in pEFE cells. Indeed, immunocytochemistry (ICC) assay using anti-ALMS1 antibodies confirmed ALMS1 protein localization to the centrioles in control hNDFs, while ALMS1 protein was completely absent in the proband pEFE-derived dermal fibroblasts, indicating a null effect (Fig. 3B).

### Novel ALMS1 variant alters the functional phenotype of pEFE fibroblasts

To examine the functional impact of the novel *ALMS1* variant at the cellular level, we first examined cilia morphology in pEFE dermal fibroblasts compared to control hNDFs using electron microscopy (EM). We observed a unipolar organization of thin, over-branched, microvilli, nonmotile finger-like protrusions from the surface of cells that function to increase the adhesion, the cell surface area, and the efficiency of absorption, which was more prominent in pEFE cells compared to control hNDFs (Fig. 3C). Next, we examined the impact on cellular proliferation over time using an automated proliferation assay performed on an xCELLigence RTCA SP instrument. We observed decreased proliferation index in pEFE fibroblasts, compared to hNDFs (Fig. 3D, E). FACS analysis revealed no change in G2/M phase in pEFE fibroblast compared to control (Supplemental Fig. 2). Then, we examined the impact on cellular migration over time using the xCELLigence RTCA DP instrument and observed increased migration activity in pEFE fibroblasts compared to the control hNDFs (Fig. 3F, G). Together, the novel *ALMS1* variant altered the functional phenotype and cellular physiology of pEFE fibroblasts.

### Novel ALMS1 variant impact on pEFE proband heart

**Fig. 4:**
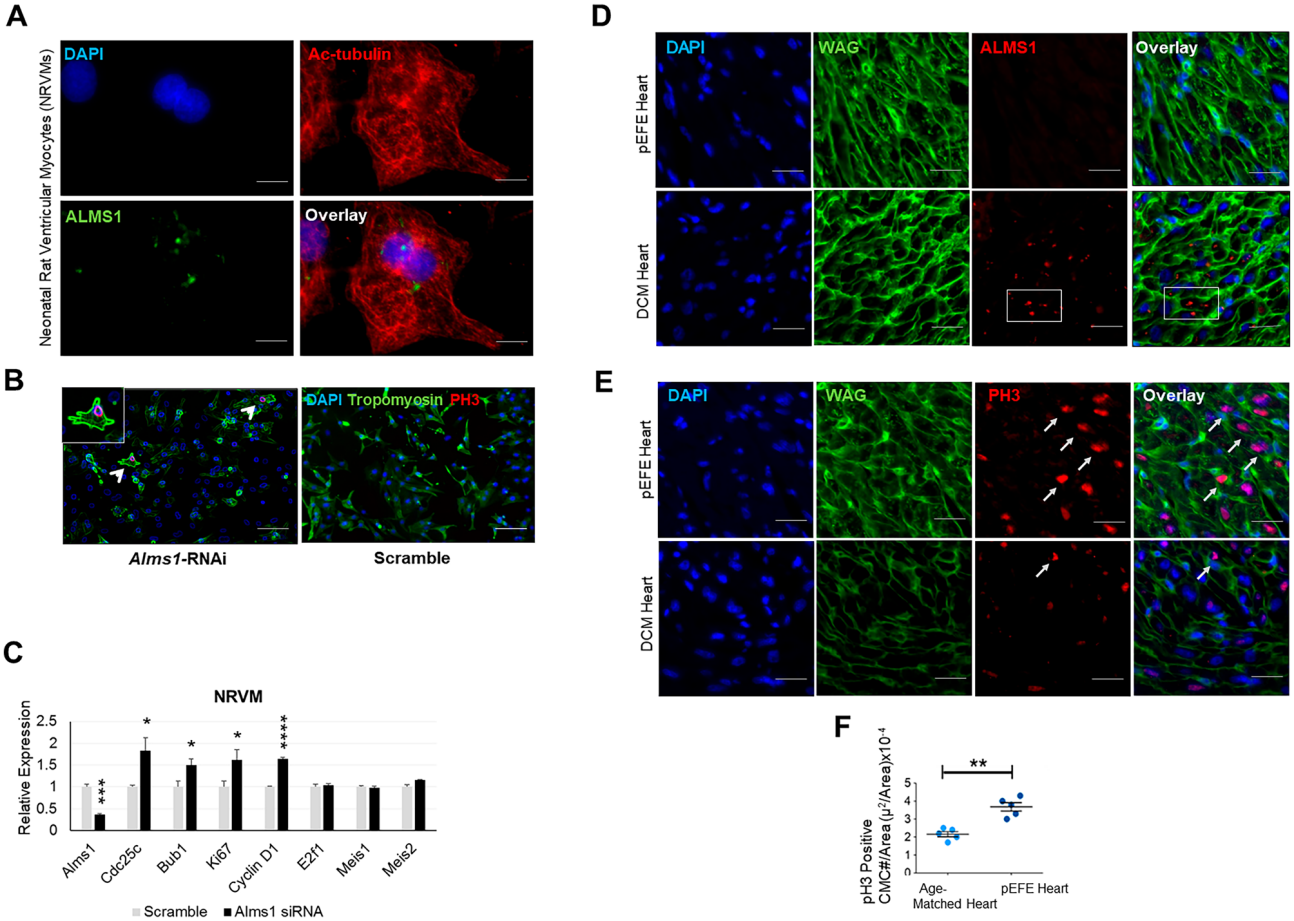
The novel *ALMS1* variant abolishes ALMS1 protein expression and induces cardiomyocyte proliferation in pEFE heart. (**A**) Representative fluorescence microscopy images of neonatal rat ventricular myocytes (NRVMs) show ALMS1 localization at the centromeric poles of the mitotic spindle. (**B**) Representative fluorescence microscopy images of phospho-Histone 3 (pH3)-stained NRVMs depict enhanced proliferation activity in Alms1-depleted neonatal cardiomyocytes. (**C**) qRT-PCR analysis of Alms1-depleted NRVMs. *N* = 3 biological replicates per condition. *p value < 0.05, ***p value < 0.005, ****p value < 0.001. (**D**) Representative fluorescence microscopy images of ALMS1 IHC in pEFE heart tissue and an age-matched proband with DCM. (**E**) Representative fluorescence microscopy images of pH3-stained heart sections demonstrate increased proliferation activity (arrows) in pEFE heart compared to age-matched DCM heart. (**F**) Quantitative analysis of pH3-positive cardiomyocytes in pEFE heart compared to DCM heart (*n* = 3 sections per heart, 5 surface areas per section)

As previously stated, few reports have implicated homozygous *ALMS1* mutations in an extremely rare form of neonatal cardiomyopathy associated with AS and characterized by prolonged mitotic window of postnatal cardiomyocytes, hence termed “mitogenic cardiomyopathy” [18, 19]. Furthermore, mice with *Alms1* mutations exhibit delayed exit from active cell cycle progression [20], supporting a functional role for ALMS1 in regulating postnatal cardiomyocyte maturation. Based on these reported observations, we sought to determine the pathogenic impact of the mutant ALMS1 in pEFE heart. First, we determined that ALMS1 protein is also localized at the centrosomic poles (centrioles) in neonatal cardiomyocytes by using primary cultured neonatal rat ventricular myocytes (NRVMs) isolated from the neonatal rat heart (Fig. 4A). Next, we confirmed that ALMS1 loss is sufficient to extend the proliferative activity of postnatal cardiomyocytes by using small interfering RNA (siRNA)-mediated inhibition of *Alms1* in primary cultured NRVMs. We observed increased mitotic activity in Alms1-deficient myocytes, as indicated by increased number of phospho-Histone H3 (pH3)-positive cardiomyocytes and upregulation of the mitotic marker genes, including *Ki67* and *Cdc25c*, compared to control scramble (Fig. 4B, C). These results are consistent with previous studies [18–20], indicating that ALMS1 regulates neonatal cardiomyocyte proliferation. Then, we confirmed that ALMS1 expression at the centrosomes was absent in histopathological sections obtained from the proband pEFE heart, but appeared normal in the age-matched DCM heart with a pathogenic mutation in *TNNT2* (Fig. 4D). Finally, we evaluated cardiomyocyte proliferation activity in proband pEFE heart sections, using pH3 immunohistochemistry (IHC) analysis, and revealed increased pH3-positive cardiomyocytes in pEFE heart compared to the age-matched DCM heart (Fig. 4E, F). Together, in agreement with other reported cases of AS-associated mitogenic cardiomyopathy, the novel *ALMS1* variant abolished ALMS1 protein expression and localization at the centrioles and delayed cardiomyocyte proliferation arrest in pEFE heart. However, how the novel ALMS1 variant leads to pEFE phenotype remains unclear.

### Novel ALMS1 variant alters global transcriptome signature in pEFE fibroblasts

Having established the causal role of the novel *ALMS1* variant [c.1943delA] in pEFE, we aimed to gain further insights into the mechanisms that underlie ALMS1 function in endocardial fibroelastosis process by evaluating the global impact of ALMS1 loss on transcriptome programming of proband pEFE cells that carry the mutation. We systematically analyzed pEFE dermal fibroblast-derived RNA-seq datasets using total RNA obtained from three independent biological replicates. In addition, three independent hNDF replicates were subjected to the same RNA-seq protocol as we previously described [26, 27].

**Fig. 5:**
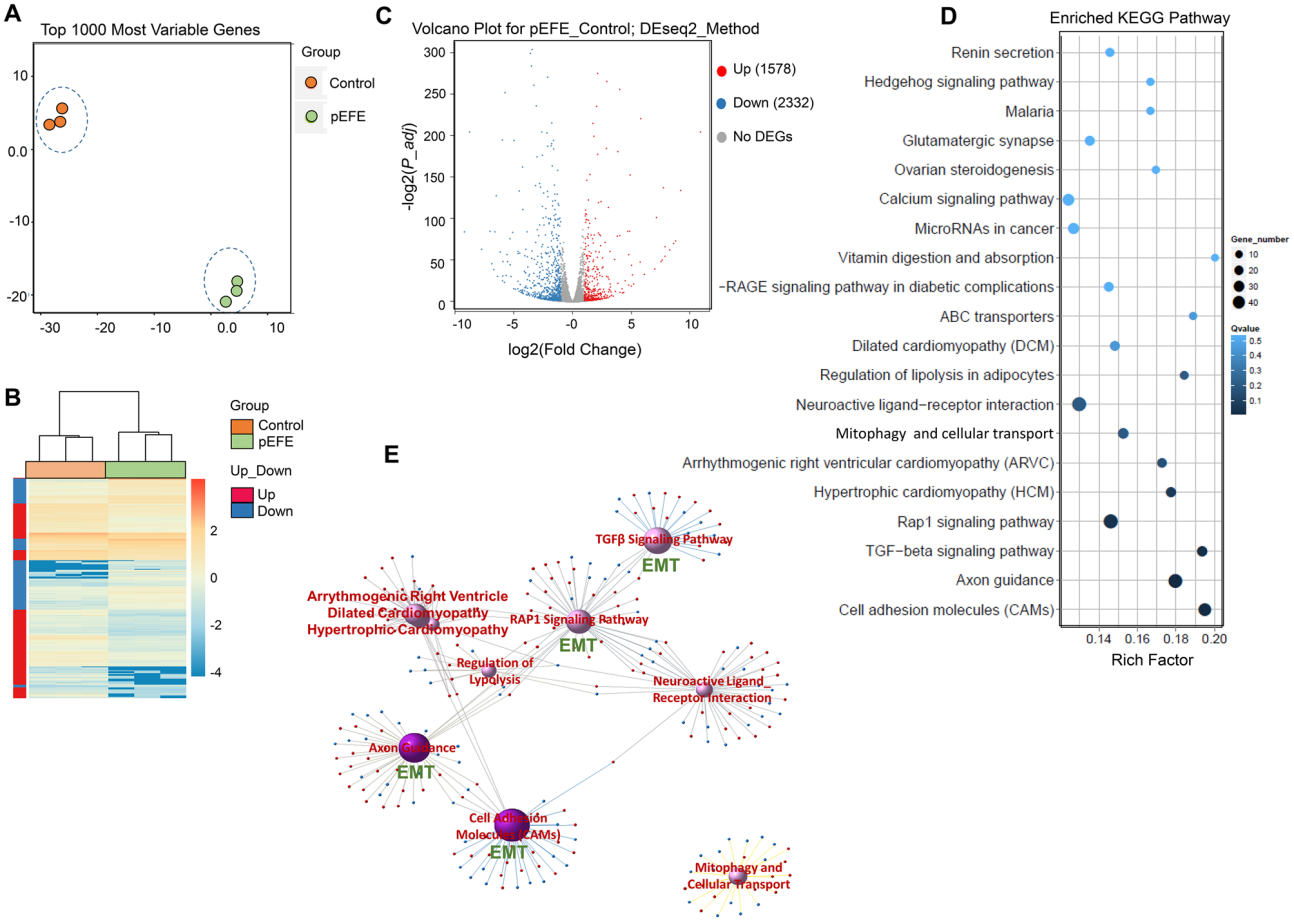
The novel *ALMS1* variant alters the molecular signature of pEFE fibroblast. (**A**) Principal component analysis of top 1000 varied genes in RNA-seq-derived data from pEFE fibroblasts compared to control (hNDFs). *N* = 3 biological replicates per group. (**B**) Transcriptome landscape of pEFE fibroblasts compared to control (hNDFs). *N* = 3 biological replicates per group. *X* axis represents the sample. *Y* axis represents the differentially expressed genes (DEGs). The color represents the log10 transformed gene expression level. Dark color means high expression level while light color means low expression level. (**C**) Volcano plot of significant DEGs in pEFE fibroblasts vs control (hNDFs) using DEseq2 method. Red: upregulated, blue: downregulated. (**D**) KEGG pathway analysis of DEGs in pEFE proband fibroblasts vs control (hNDFs). *X* axis represents enrichment factor. *Y* axis presents pathway name. The color indicates the *q*-value (high: white, low: blue). Lower *q*-value indicates more significant enrichment. Size of the dot indicates DEG number. Rich Factor refers to the value of enrichment factor. Larger value indicates more significant enrichment. (**E**) KEGG-DEGs in pEFE fibroblasts vs control (hNDFs) relationship network. Purple balls represent the top ten enriched pathways. The dark color indicates a significant enrichment (*Q* value < 0.01), while the light color indicates enrichment that is not significant. Larger ball indicates higher degree of enrichment. The red and blue dots in each network represent the upregulated and downregulated genes, respectively

Principal component analyses of the top 1000 varied genes showed that transcripts from pEFE and control fibroblasts formed distinct clusters (Fig. 5A). Likewise, expression heatmap across the entire transcriptome revealed distinct molecular signatures for pEFE and control samples (Fig. 5B), indicating that the variation pattern was consistent across the two methods. In total, 8970 protein coding genes were expressed at ≥ 3 RPKM in at least three samples (3 biological replicates) with coefficient of variation (CV) exceeding 0.2. Of these expressed genes, 3910 genes exhibited significant differential gene expression (DGE) in pEFE versus control at Benjamini-Hochberg (B-H)-adjusted *P* value less than 0.05 (Fig. 5C). Together, these findings indicate significant impact of *ALMS1* perturbation on global transcriptome signature in pEFE fibroblasts compared to the control hNDF cells.

### Novel ALMS1 variant activates epithelial mesenchymal transition (EMT) and induces TGFβ signaling

Gene ontology analysis of the differentially expressed genes was performed using Gene Set Enrichment Analysis (GSEA) [30]. EMT, a process that converts epithelial cells to mobile mesenchymal cells and plays an important role in cardiac development [31], was predominantly enriched in the upregulated genes. Altogether, 175 EMT-related genes exhibited significant induction (Supplemental Tables 3–5), including myofibroblast cell surface marker genes (EDA, POSTN), key transcription regulators of EMT (ID1, ID2, SNAIL), and major signaling players of EMT (FZD8, TGFβ). Additional features of EMT were also reflected by upregulated genes involved in ECM degradation (MMP14, LOXL1) and increased cell motility and migration ability (FGFR2, PRKG2), and downregulated genes involved in apical junction assembly (JUP, CDSN) and P53 signaling (PIDD1, CASP1). In contrast, cell cycle genes were downregulated in pEFE fibroblast compared to control. Together, the molecular signature of pEFE fibroblasts is consistent with an EMT state and a migratory, non-proliferative, cellular phenotype.

To annotate the signaling pathways enriched in the differentially expressed genes, we used Kyoto Encyclopedia of Genes and Genomes (KEGG) pathway enrichment analysis and found cellular adhesion, exon guidance, and TGFβ and RAP1 signaling as the top enriched functional pathways that were also interconnected by sharing several overlapping genes based on the DGE-KEGG network relation analysis (Fig. 5D, E). For example, genes involved in EMT significantly overlapped with cell adhesion molecule and TGFβ pathways. In particular, we identified BMPR1B, a known member of the bone morphogenetic protein (BMP) family of transmembrane serine/threonine kinases that has been associated with left ventricular mass [32]. The ligands of this receptor are members of the BMPs and TGF-β superfamily. Consistently, by performing upstream analysis using IPA (Ingenuity Pathway Analysis) of all genes within the connected networks, we revealed TGFβ as the top upstream regulator with remarkable induction of TGFβ downstream signaling mediators, including *BMPR1B*, *ID1-4*, *SMAD9*, *INHBB*, *CHRD*, and *BMP6*, many of which are key players of EMT during embryonic development and cardiogenesis [31] (Supplemental Tables 3–5). Together, our findings suggested TGFβ-mediated activation of EMT program in the proband pEFE fibroblasts.

**Fig. 6:**
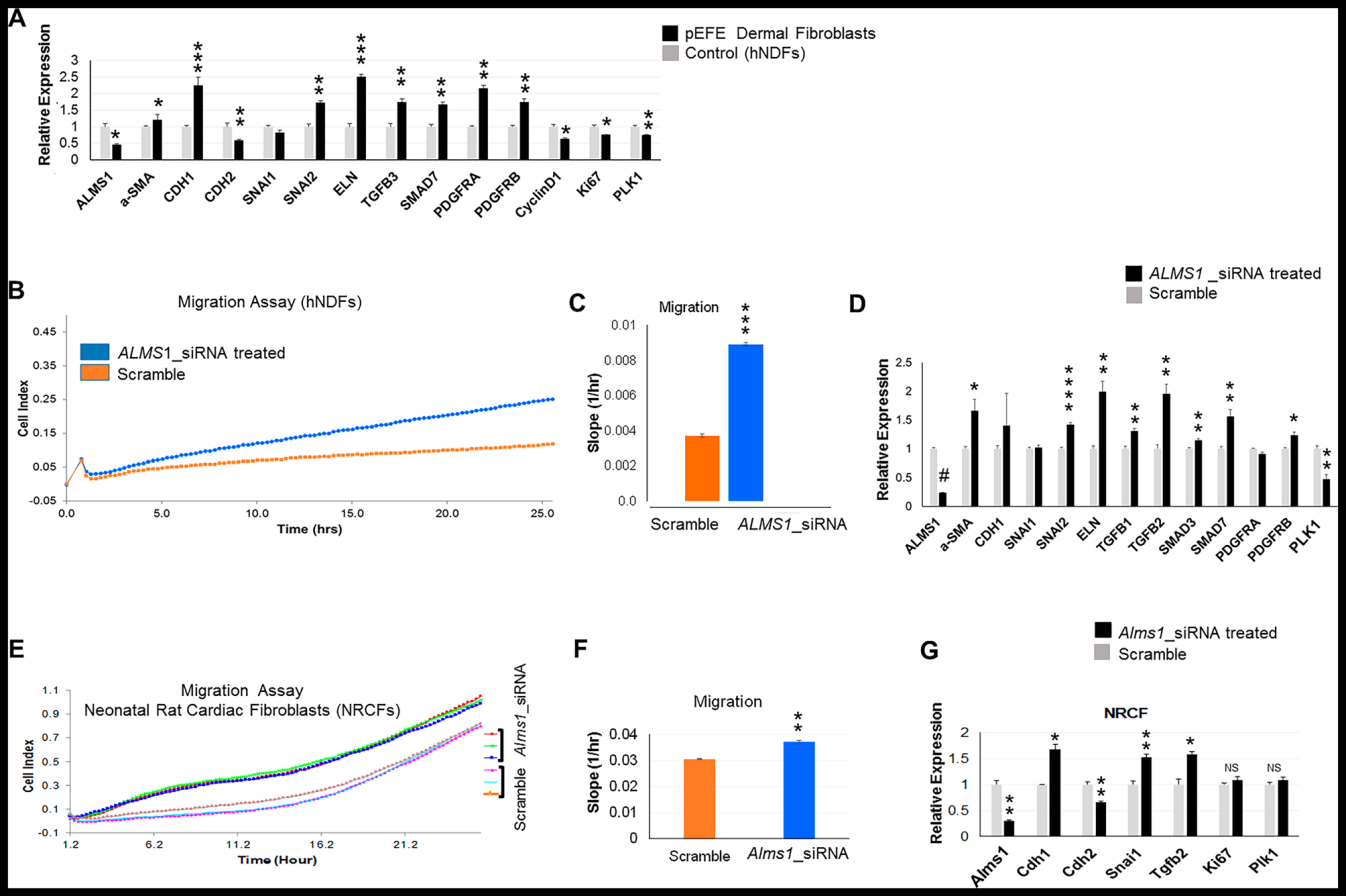
ALMS1 loss induces migration and activates epithelial to mesenchymal transition (EMT) program in neonatal dermal fibroblasts and neonatal cardiac fibroblasts. (**A**) qRT-PCR analysis of EMT and proliferation marker genes in pEFE proband fibroblasts compared to control (hNDFs). *N* = 3 biological replicates per group. *p value < 0.05, **p value < 0.01, ***p value < 0.005. (**B**) Migration assay of *ALMS1* siRNA-treated vs scramble-treated hNDFs exhibited enhanced migration activity as demonstrated by higher cellular index over time. The migration assay was performed using an xCELLigence RTCA DP instrument over a 25-h period. 10,000 cells per well were seeded into the upper chamber of CIM-Plate16 . *N* = 5 biological replicates per group. (**C**) Quantitative analysis of fibroblast migration assay shown in (**B**). (**D**) qRT-PCR analysis of EMT and proliferation marker genes in *ALMS1*-depleted hNDF compared to control scramble. Inhibition of *ALMS1* expression in *ALMS1* siRNA-treated hNDF was confirmed as well. *N* = 5 biological replicates per condition. *p value < 0.05, **p value < 0.01. ***p value < 0.005. ****p value < 0.001. #*p* value < 2e-07. (**E**) Migration assay of Alms1-depleted NRCFs compared to scramble control. Alms1-depleted NRCFs exhibited enhanced migration activity as demonstrated by higher cellular index over time. The migration assay was performed using an xCELLigence RTCA DP instrument over a 25-h period. 20,000 cells per well were seeded into the upper chamber of CIM-Plate16 . *N* = 3 biological replicates per condition. (**F**) Quantitative assessment of NRCF migration assay shown in (**E**). **p value < 0.01. (**G**) qRT-PCR analysis of EMT and proliferation marker genes in Alms1-depleted NRCFs compared to control scramble. Inhibition of Alms1 expression in Alms1 siRNA-treated NRCFs was confirmed as well. *N* = 3 biological replicates per condition. *p value < 0.05, **p value < 0.01

To validate these findings, we examined the impact of the novel *ALMS1* variant on selected EMT marker genes in primary cultured pEFE fibroblasts compared to control hNDFs using qRT-PCR. Indeed, TGFβ was induced along with the transcription factor *SNAIL*, the ECM marker *ELN* as well as the myofibroblast marker gene *aSMA*. Consistently, *CDH1*, a key signaling mediator for the induction of EMT cascade, was significantly upregulated, while *CDH2* was downregulated. Finally, cell cycle markers (*Ki67* and *Plk1*) were suppressed (Fig. 6A). Together, the data support that ALMS1 loss leads to EMT induction, potentially via the activation of TGFβ signaling. To futher confirm the impact of ALMS1 loss, we treated primary cultured hNDF cells with ALMS1-targeting small interfering RNAs (siRNAs). Indeed, ALMS1-depleted hNDFs exhibited significantly enhanced migration and upregulation of EMT marker genes, replicating the changes seen in pEFE fibroblasts (Fig. 6B–D).

### Novel ALMS1 mutation induces EMT in neonatal cardiac fibroblasts

Current evidence suggests a role for EMT in EFE [33, 34]. Our transcriptome analysis suggested that *ALMS1* loss altered the physiological proprieties (enhanced migration) and induced EMT in pEFE fibroblasts potentially via activating TGFβ signaling. We sought next to determine whether ALMS1 regulates EMT in cardiac fibroblasts by performing siRNA-mediated knockdown of Alms1 in primary cultured NRCFs. Remarkably, the cells exhibited enhanced migration and induced EMT marker genes, including the *Snail1*, *Tgfb*, and *Cdh1* (Fig. 6E–G). Importantly, unlike neonatal cardiomyocytes, NRCFs remained quiescent and did not exhibit increased proliferation activity as demonstrated by proliferation marker genes (*Plk1* and *Ki67*) expression (Fig. 6G). Together, the data demonstrate EMT induction and *Tgfb* activation in Alms1-deficient neonatal cardiac fibroblasts. These findings replicate the changes observed in the proband pEFE dermal fibroblasts and ALMS1-deficient hNDFs, and correspond to a known role of cilia-mediated signaling in driving EMT process in cardiac development and fibrosis in response to cardiac injury [35].

### Further surveillance for Alstrom syndrome and patient care

**Fig. 7:**
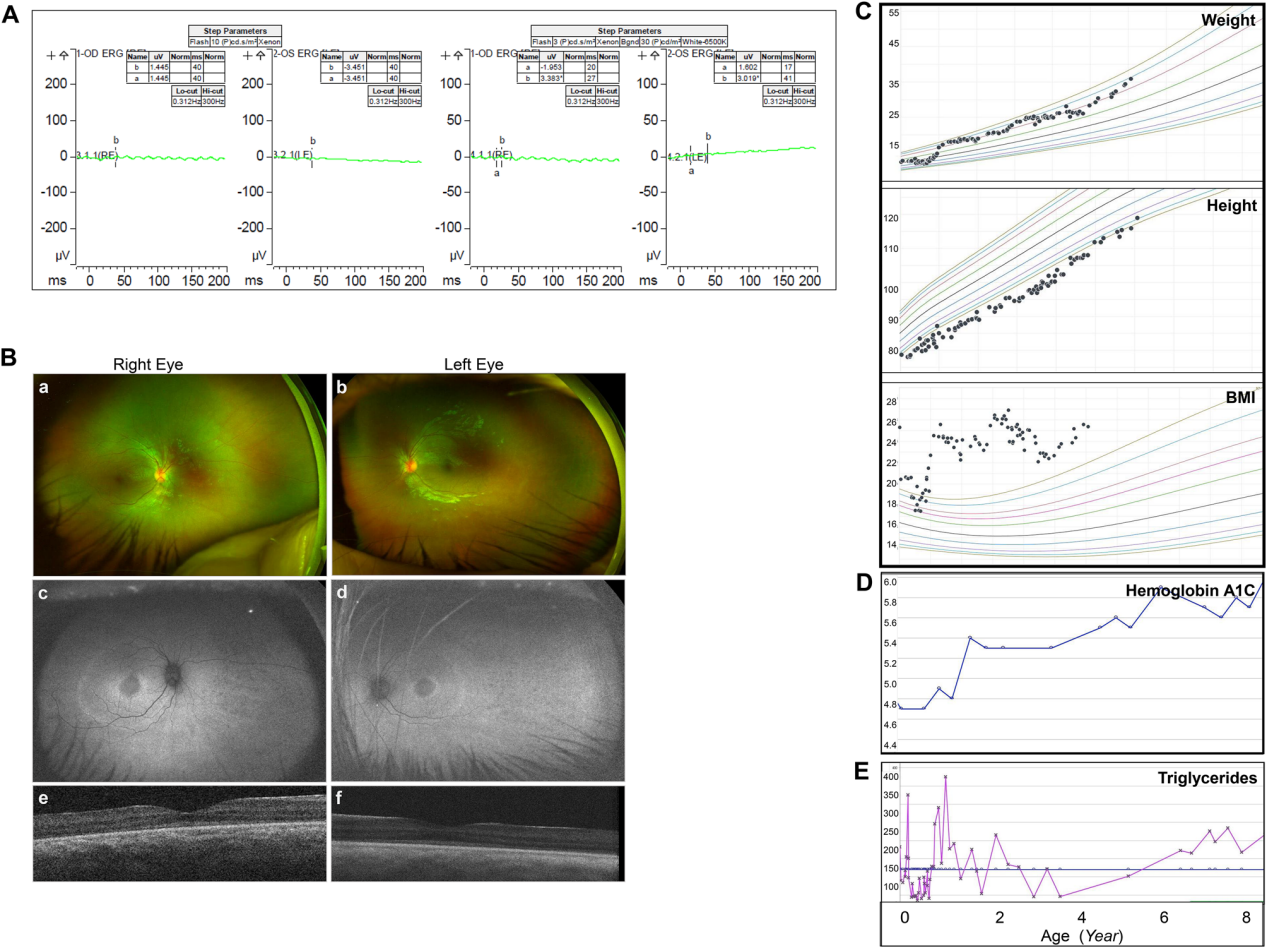
The pEFE proband develops other manifestations of Alstrom syndrome. (**A**) Electroretinography (ERG) results of the pEFE proband at 7 years of age indicate visual impairment. (**B**) Retinal findings in the pEFE proband. (**a**, **b**) Optos widefield pseudocolor fundus images show mild pigmentary changes in the retinal periphery of the right (**a**) and left (**b**) eyes. (**c**, **d**) Fundus autofluorescence imaging highlights the findings associated with ALMS1-associated retinal dystrophy including central hypoautofluorescence, perifoveal and perimacular hyperautofluorescent rings, and peripheral mottled hyper/hypoautofluorescence in the right (**c**) and left (**d**) eyes. (**e**, **f**) Spectral-domain optical coherence tomography shows ellipsoid zone attenuation with more pronounced loss in the perifoveal region in the right (**e**) and left (**f**) eyes. (**C**) Growth charts of the pEFE proband indicate short stature and early onset of obesity (rapid increase of BMI at 2–4 years of age). (**D**) The pEFE proband presents increased hemoglobin A1C. (**E**) The pEFE proband presents increased serum triglyceride levels

According to the diagnostic criteria from Marshall et al. [15], detection of the novel *ALMS1* variant [c.1943delA] suggested the genetic diagnosis of AS. We provided detailed genetic consultation about AS to the family and monitored our pEFE proband closely for potential multisystem involvement, in addition to the regular monitoring and management by the primary cardiologist and heart transplantation clinic. Bilateral nystagmus, ptosis, and photophobia were documented at 6–12 months of age. Visual impairment was diagnosed at 1 year of age. Standard electroretinography (ERG) at 7 years of age showed absence of scotopic, photopic, maximal-combined, and flicker responses, consistent with severe pan-retinal abnormalities of both rod- and cone-mediated retinal functions (Fig. 7A). Retinal imaging revealed bilateral retinal dystrophy (Fig. 7B). Short stature, increased weight gain, and early-onset obesity were observed at 2–3 years of age associated with elevated triglycerids, acanthosis nigricans, and increased hemoglobin A1C (HbA1C) suggesting insulin resistance (Fig. 7C–E). Hearing evaluation, thyroid function tests, blood sugar levels, serum lipid profile, liver function tests, serum creatinine, abdominal ultrasound, and blood chemistry remained normal. The patient maintained normal bilateral audiology and psychomotor functions.

## Discussion

In this report, we describe the unique pathological finding of pEFE compared to DCM. We also elucidate novel genetic contribution to pEFE etiology involving a known ciliopathy gene. By complementing trio-based WES with RNA-seq of patient-derived fibroblasts, our integrative genomic analysis allowed the identification of a novel pathogenic variant. Herein, we report our novel discovery of *ALMS1* as a causal gene underlying pEFE pathogenesis. To our knowledge, this is the first case of pEFE associated with Alstrom syndrome (AS), a rare recessive ciliopathy.

Our report expands the phenotypic and genetic features of pEFE and AS. The patient reported here initially presented with a unique form of isolated neonatal cardiomyopathy that could lead to early death [7]. Subsequent WES analysis revealed the genetic diagnosis of AS. Having survived heart transplantation at 1 year of age, surveillance for multi-organ involvement and targeted interventions can help improve long-term outcomes for this patient [21]. Since *ALMS1* is the only gene in which recessive mutations are known to cause AS manifestations, including cardiomyopathy [14–22], we ascertained the inheritance pattern, leading to appropriate counseling. Therefore, we propose utilizing WES as the first diagnostic tool to establish an early diagnosis for neonatal cardiomyopathies, *ALMS1* should be added to the cardiomyopathy gene panel. Since the cardiomyopathy in Alstrom syndrome, in this case at least, is pEFE, patients with infantile cardiomyopathy/pEFE should be monitored closely for developing features of AS.

The giant ALMS1 protein, predicted to comprise 4,169 amino acids, is widely expressed and localized to the centrosomes and basal bodies of ciliated cells of different organs [28, 29]. *ALMS1* loss of function has been linked to defects in primary cilium formation, positioning, and maintenance, leading to AS being classified as a ciliopathy [14, 15]. Reportedly, the ALMS motif is the only region that shares obvious sequence similarity with other human proteins and confers an evolutionarily conserved function in targeting ALMS1 protein to the centrioles [29]. Consistent with previous reports, the ALMS1 protein was absent in the centrioles of the proband pEFE fibroblasts and heart tissue sections, confirming a deleterious effect of the novel *ALMS1* variant on ALMS1 protein translation and localization. Although the exact functions of the ALMS1 protein are yet to be fully revealed, it has been demonstrated to play a role in cell cycle regulation [18–20]. Consistently, proliferation activity was enhanced in pEFE heart tissue compared to DCM. Likewise, Alms1-depleted cardiomyocytes exhibited a modest increase in their mitotic activity. On the contrary, Alms1-depleted NRCFs, ALMS1-depleted hNDFs, and dermal fibroblasts from pEFE proband exhibited reduced proliferative capacity. However, they demonstrated enhanced migration abilities when compared to their control counterparts. Together, these conflicting phenotypic properties in ALMS1-depleted fibroblasts, in contrast to cardiomyocytes, may suggest cell type-specific role for ALMS1 protein in the heart and potentially other organs.

Alms1-deficient mice have been studied and the initial formation of primary cilia appeared normal in all three mouse models reported to date. However, age-dependent loss of primary cilia was observed in two of these mouse models [Alms1^L2131X/L2131X^ and Alms1^foz/foz^] [17]. Likewise, the primary cilia formation seems to be unaffected in reported AS patient fibroblasts in vitro [36]. Consistent with these reports, primary cilia formation appeared normal in our pEFE proband fibroblasts. The observed unipolar localization and confluent networking of branching cilia are potentially consistent, in part, with the observed enhanced cellular motility and migration properties. Whether these differences in the cilia-loss occurrence/timing, seen in previous murine studies, are mutation specific or cell type specific remains to be interrogated.

Cilia play essential roles throughout cardiac development. During cardiogenesis, primary cilia are present on the majority of heart cell types acting as cellular sensors for signal transduction that control cardiac myocytes and fibroblast differentiation [37–39]. Deletions in cilia-related genes have been implicated in congenital heart defects such as heterotaxy, which may manifest clinically as an isolated disorder or associated with other features of situs inverses [23, 37, 38]. In the case of AS, dilated cardiomyopathy during childhood is a common feature [21, 22]. Meanwhile, only few reports have described an extremely rare feature of AS called “mitogenic cardiomyopathy” that is characterized by persistent mitotic activity in neonatal cardiomyocytes [18–20]. Importantly, like pEFE, AS-associated mitogenic cardiomyopathy can be lethal during infancy, before other clinical features of AS normally manifest. However, in contrast to the unique pEFE phenotype, no abnormalities in the endocardium have been described in mitogenic cardiomyopathy cases that are reported thus far. Indeed, our report provides the first link between ciliopathy and pEFE.

How ALMS1 contributes to pEFE remains to be fully revealed. The involvement of the endocardium is a distinct, yet mysterious, phenomenon in pEFE hearts. Unlike secondary EFE that associates with hypoplastic left heart or myocarditis, no structural defect is seen in pEFE cases, meriting further investigations. Hence, unusual human phenotypes offer the opportunity to understand disease pathogenesis, if the cause can be determined. Having identified a causal homozygous *ALMS1* variant in pEFE, the availability of dermal fibroblasts derived from the pEFE proband allowed us to examine the broader impact at the molecular and cellular levels by performing unbiased transcriptome analysis of the patient cells that harbor the *ALMS1* [c.1943delA] mutation, using hNDFs as control. EFE is a specialized form of cardiac fibrosis that involves excessive fibroblast-mediated deposition of elastin (ELN) and collagen fibers, predominantly, in the subendocardium layer of the left ventricle [8–11]. Consistently, *ALMS1*-deficient fibroblasts from the pEFE proband exhibited significant upregulation of several ECM constituents compared to hNDF (Supplemental Table 5).

Different cardiac lineages arise from one or more EMT waves during cardiogenesis. Errors of the EMT process, including ciliopathies, may lead to cardiac defects and fibrosis [31, 32]. An epithelial cell must lose epithelial cell polarity and acquire a motile phenotype to undergo EMT. The Snail proteins are key EMT-inducing transcription factors activated by EMT-inducing stimuli such as TGFβ. In turn, Snail suppresses adhesion protein genes such as E-cadherin and upregulates the cytoskeletal proteins leading to cytoskeletal reorganization. Furthermore, it stimulates the expression of matrix metalloproteases (MMPs) that break down the ECM and facilitate cellular migration. In agreement, pEFE fibroblast transcriptome profile uncovered clear activation of EMT including reprogramming of fibroblasts to migratory cells exhibiting myofibroblasts marker genes, along with the upregulation of MMPs and suppression of apical junction genes, likely triggered by TGFβ signaling activation. However, the cellular origin of pEFE fibrosis remains unclear. It was previously suggested that the EFE fibroblasts arise from postnatal endocardial cells that undergo an aberrant endothelial-to-mesenchymal transition (End-MT) [33]. However, using an EFE-like mouse model with hemodynamically unloaded heart and multiple genetic lineages tracing approach, a more recent study showed that the epicardium-derived mesenchymal cells (Epi-MCs) serve as the major source for EFE fibroblasts [34]. Further investigations to determine the cellular origins of EFE fibroblasts are essential to delineate EFE pathogenesis. Moreover, the role of cilia signaling pathways that govern Epi-MCs expansion needs to be fully addressed.

Finally, consistent with the previous transcriptomic analysis of *ALMS1* mutants in other species [40, 41], RNA-seq data derived from pEFE fibroblasts revealed induced axon guidance and increased secretory activity, endosomal transport, and cellular trafficking. Furthermore, retinol, leptin, and insulin-related pathways were dysregulated (Supplemental Table 6). Since obesity, insulin resistance, and related metabolic defects are cardinal characteristics of AS [42], it is quite interesting to observe that the insights gained from transcriptome analysis of pEFE fibroblasts recapitulate physiological defects of AS patients and AS animal models. These conserved effects underscore the utility of integrated genomics analysis (exome/transcriptome) in patient-derived cells to interpret human phenotypes.

Several limitations need to be acknowledged in our study. Our functional studies are limited to in vitro analysis. Further mechanistic studies of Alms1-mediated ciliary signaling in Alms1 mouse models are necessary to fully elucidate the mechanisms that underlie pEFE fibrosis and neonatal cardiomyocyte proliferation in AS. It would be essential to characterize ALMS1 function and potential downstream mediators that might impact neonatal heart maturation and to identify disease-specific therapeutic targets.

## Supplementary information

Below is the link to the electronic supplementary material.

Supplementary file1 (XLSX 27 KB)

Supplementary file2 (PDF 162 KB)

Supplementary file3 (PDF 226 KB)

## Data Availability

The datasets used and analyzed during the current study are available from the corresponding author upon reasonable request***.*** Upon acceptance of this manuscript, WES and RNA-seq datasets will be deposited in the NCBI’s Gene Expression Omnibus repository under Neonatal Heart Maturation SuperSeries GSE85728 (http://www.ncbi.nlm.nih.gov/geo/query/acc.cgi?acc=GSE85728/) or according to the journal instruction.

## Abbreviations

pEFE: Primary endocardial fibroelastosis
DCM: Dilated cardiomyopathy
WES: Whole exome sequencing
RNA-seq: RNA sequencing
EMT: Epithelial mesenchymal transition
EM: Electron microscopy
AS: Alstrom syndrome
NRVM: Neonatal rat ventricular myocyte
NRCF: Neonatal rat cardiac fibroblast
hNDF: Human neonatal dermal fibroblast
