# Supplementary material for: Recessive ciliopathy mutations in primary endocardial fibroelastosis: a rare neonatal cardiomyopathy in a case of Alstrom syndrome
Source: J Mol Med (Berl). 2021 Aug 13;99(11):1623–38. doi: 10.1007/s00109-021-02112-z (PMC8541947; doi:10.1007/s00109-021-02112-z)
Supplement: Supplementary file 2 — Supplementary file2 (PDF 162 KB) [file 109_2021_2112_MOESM2_ESM.pdf]

SUPPLEMENTAL MATERIALS

Integrated Genomics Analysis Identifies Recessive Ciliopathy Mutations in Primary Endocardial Fibroelastosis: a Rare Neonatal Cardiomyopathy in a Case of Alstrom Syndrome

AUTHORS AND AFFILIATIONS

Yan Zhao[1-3], Lee-kai Wang[4], Ascia Eskin[5], Xuedong Kang[1-3], Viviana M. Fajardo[1], Zubin Mehta[1-3], Stacy Pineles[6], Ryan J. Schmidt[7], Aaron Nagiel[8,9], the UCLA Congenital Heart Defects BioCore Faculty[$], Gary Satou[1], Meena Garg[1], Myke Federman[1], Leigh C. Reardon[1,10], Steven L. Lee[1], Reshma Biniwale[1,11], Wayne W. Grody[1,12], Nancy Halnon[1], Negar Khanlou[12], Fabiola Quintero-Rivera[13], Juan C. Alejos[1], Atsushi Nakano[15],Gregory A. Fishbein[12], Glen S. Van Arsdell[1,11], Stanley F. Nelson[1,4,5], Marlin Touma[1-3,14,15.]

1. Department of Pediatrics, David Geffen School of Medicine, University of California Los Angeles, Los Angeles, CA.
2. Neonatal/Congenital Heart Laboratory, Cardiovascular Research Laboratory, University of California Los Angeles, Los Angeles, CA.
3. Children’s Discovery and Innovation Institute, Department of Pediatrics, David Geffen School of Medicine, University of California Los Angeles, Los Angeles, CA.
4. Institute for Precision Health, David Geffen School of Medicine, University of California Los Angeles, Los Angeles, CA.
5. Department of Human Genetics, David Geffen School of Medicine, University of California Los Angeles, Los Angeles, CA.
6. Department of Ophthalmology, David Geffen School of Medicine, University of California Los Angeles, Los Angeles, CA.
7. Department of Pathology and Laboratory Medicine, Children’s Hospital Los Angeles, Los Angeles, CA.
8. The Vision Center, Department of Surgery, Children’s Hospital Los Angeles, Los Angeles, CA.
9. Roski Eye Institute, Department of Ophthalmology, University of Southern California. Los Angeles, CA.
10. Ahmanson/UCLA Adult Congenital Heart Disease Center. Department of Medicine, David Geffen School of Medicine, University of California Los Angeles, Los Angeles, CA.

11. Department of Cardiothoracic Surgery, David Geffen School of Medicine, University of California Los Angeles, Los Angeles, CA.
12. Department of Pathology and Laboratory Medicine, David Geffen School of Medicine, University of California Los Angeles, Los Angeles, CA.
13. Department of Pathology and Laboratory Medicine, School of Medicine, University of California Irvine, Irvine, CA.
14. The Molecular Biology Institute, David Geffen School of Medicine, University of California Los Angeles, Los Angeles, CA.
15. Eli and Edythe Broad Stem Cell Research Center, David Geffen School of Medicine, University of California Los Angeles, Los Angeles, CA.

CORRESPONDENCE

Marlin Touma, MD, PhD,

Department of Pediatrics,

David Geffen School of Medicine, University of California, Los Angeles

675 Charles E. Young Dr S, 3762 MacDonald Research Laboratories,

Los Angeles, CA 90025. USA.

Tel: 310-825-6478 / Fax: 310-267-0154 /Email: mtouma@mednet.ucla.edu

---

[$]**.** The UCLA Congenital Heart Defect BioCore Faculty: Marlin Touma, Glen Van Arsdell, Nancy Halnon, Juan Alejos, Leigh Reardon, Amy Speirs, Meena Garg, Myke Federman, Paul Finn, Fabiola Quintero-Rivera, Wayne W. Grody, Stanley F. Nelson, Yibin Wang.

SUPPLEMENTAL METHODS

Skin and Muscle Biopsy, Heart Tissue Specimens: Using betadine, then alcohol wipe, the skin was prepped and draped in a sterile fashion. For local anesthesia, approximately 1 ml Lidocaine 1% was injected intradermally and 3mm diameter biopsy punch was performed through the skin. The biopsy sample was placed in DMEM culture media and transported to the lab for adherent primary dermal fibroblast culture following standard protocol. The control human neonatal dermal fibroblasts (hNDFs) were purchased from ATCC. Heart tissue sections were obtained from UCLA clinical pathology laboratory. All samples were de-identified and coded upon procurement.

Whole Exome Sequencing (WES): Genomic DNA was extracted using standard methods (Purelink Genomic DNA Mini Kit, Invitrogen) at the UCLA Congenital Heart Defect-BioCore. Library preparation, sequencing and data analysis were performed at the CCRD (California Center for Rare Disease) and the UCLA Clinical Genomics Center, using the CLIA (Clinical Laboratory Improvement Amendments) and CAP (College of American Pathologists) -validated protocols. Genomic DNA (3 µg) from the proband and parents was subjected to library preparation and exome capture following the Agilent SureSelect Human All Exon 50 Mb Illumina Paired-End Sequencing Library Prep Protocol. Sequencing was performed on an Illumina HiSeq2000 as a 50 bp paired-end run. For each sample, approximately 200 million independent paired reads were generated for an average coverage of 140X of RefSeq protein coding exons and flanking introns (+2 bp) with at least 95% of these bases covered at ≥10X. The sequences were aligned to the hg19/b37 genome release using Novoalign. PCR duplicates were marked using Picard. Genome Analysis Toolkit (GATK) [**43**] was used for indel realignment and base quality recalibration. Both SNVs (single nucleotide variants) and small INDELs (insertions and deletions) were called using GATK unified genotyper. All variants were annotated using the customized VEP (variant effect predictor) engine from Ensembl. Regions of homozygosity by descent were determined using PLINK. Rare protein-altering variants were examined under different inheritance models. Rare variants with minor allele frequency <1% in public databases were retained for analysis. Variants were classified based on their pattern of inheritance, predicted consequence at the protein level, and their location within the gene. All variants were interpreted in the context of the patient's phenotype.

RNA Sequencing and Bioinformatics Analysis: RNA sequencing, data processing, and bioinformatics analysis steps follow exactly the procedures we previously described [**26, 27**]. Briefly, total RNAs were

isolated, ribosomal RNAs were depleted (RiboZero Gold, Illumina), and the resulting RNA transcripts were processed into short fragments (about 200~500nt). Strand-specific cDNA libraries were constructed using random hexamer-primed reverse transcription and subsequently were used to generate second-strand. Sequencing adaptors were ligated using the Illumina Paired-End Sample Prep Kit. Fragments of ~200 bp were isolated by gel electrophoresis to select the suitable fragments for PCR amplification. Agilent 2100 Bioanalyzer and ABI StepOnePlus Real-Time PCR System were used for quantification and evaluation of the library quality. Lastly, the libraries were sequenced according to Illumina HiSeqTM 4000 sequencer protocol (BGI-Genomics) in paired-end sequencing mode (2 x 100 nt long reads). After adaptors removal and quality control procedures, reads containing ribosomal RNA (rRNA) were removed by SOAP (Short Oligonucleotide Alignment Program) alignment. Subsequently the raw reads were aligned to reference genome using Tophat. The background noise was reduced by employing fragments abundance threshold and coverage analysis considering only those of 100 bp or longer. HISAT2 (Hierarchical Indexing for Spliced Alignment of Transcripts) was used for mapping step and transcript assembly. Normalized gene expression values were presented using mean reads per kilobase of transcript per million mapped reads (RPKM).

Differential Gene Expression (DGE) Analysis: DGE analysis was performed with expression levels normalized for gene length, library size and GC contents. DEseq2 algorithms was used to assess differential expression in log2 [normalized RPKM] values for each gene. Significant results were reported at Benjamini_Hochberg FDR ≤ 0.05. The statistical significance for DGE was assessed by Fisher's exact test. Pearson's correlation coefficients (*r*) for gene expression were calculated in R. Principal Component Analysis (PCA) was conducted using R function procomp. The top 1000 varied mRNAs based on alignment results were used to generate PCAs. The heat map function of R, which employs a hierarchical cluster algorithm, was used to draw heat map figure. The log2-transformed data were preprocessed by median centering of the data for each set and then hierarchically clustered using centered correlation as the similarity metric and average linkage as the clustering method. Volcano plot function of R was used to visualize upregulated and downregulated genes.

Other Bioinformatics and Computational Methods: Expression data analysis was supported by functional enrichment analysis, including gene ontology analysis using the Gene Set Enrichment Analysis (GSEA) suite, which calculates the global significance scores by summarizing the overall level of statistical significance of

each gene in each pathway. Pathway analysis of the differentially expressed genes was done using Kyoto Encyclopedia of Genes and Genomes (KEGG) Pathways. For upstream analysis, QIAGEN's Ingenuity Pathway Analysis (IPA) was used.

Quantitative Real-Time PCR: Total RNA was isolated from cells or tissue using an RNeasy Mini Kit (QIAGEN). For reverse transcription, one microgram of total RNA was used to generate first-strand cDNA with random primers (Applied Biosystems). Real-time PCR was performed using the SYBR Green Mix (Bio-Rad) on the CFX96 Real-time System (Bio-Rad). Data were presented as average ± SEM. Two-tailed Student's *t*-tests were used to calculate statistical significance.

Histology and Immunofluorescence Staining: Cells were fixed in 4% (v/v) formaldehyde in PBS for 20 minutes. After rinsing with PBS, the cells were incubated with 0.1% Triton X-100 in PBS for 15 minutes at room temperature and rinsed. Heart tissue was fixed in 4% (v/v) formaldehyde, embedded into paraffin, and cut into 5-μm-thick tissue sections. After deparaffinization, slides were subjected to antigen retrieval. After blocking in PBS containing 10% bovine serum albumin for 1 hour, the cells or tissue sections were incubated with primary antibodies overnight and then appropriate AlexaFluor-conjugated secondary antibodies (Invitrogen) for 1 hour. Cell nuclei were eventually counterstained by DAPI in the mounting medium (Invitrogen). Images were recorded and analyzed on a Revolve microscope (ECHO). Primary antibodies used to detect the expression of the proteins of interest are ALMS1 (Abcam), Histone H3 (Cell Signaling Technology), Phospho-Histone H3 (Millipore), Ac-alpha-Tubulin (Cell Signaling Technology), Collagen IV (Cell signaling Technology) and Pericentrin (Abcam).

Cell Culture and Small Interfering RNA Studies: Neonatal rat ventricular myocytes (NRVMs) and neonatal rat cardiac fibroblasts (NRCFs) were isolated from 2-day/old rat pups and cultured in DMEM/10% (vol/vol) FBS with antibiotics at 37°C and 5% CO2. pEFE patient-derived dermal fibroblasts and human neonatal dermal fibroblasts (hNDFs) were cultured in fibroblast growth medium (ATCC). Transient transfection of cells with siRNA (Bioland Scientific) was performed in 6-well plates using RNAiMAX (Invitrogen). Rat Alms1-siRNA (30 pmol/well) was transfected into NRVM or NRCF cultured in DMEM. For hNDFs, human ALMS1-siRNA (30 pmol/well) was used. After 48 h or 72 h of incubation, the cells were harvested.

Proliferation Assay: An xCELLigence Real Time Cell Analysis (RTCA) Single Plate (SP) instrument (Agilent) was used to monitor real-time cell proliferation. This technology works by measuring electron flow transmitted

between gold microelectrodes fused to the bottom surface of a 96-well RTCA E-plate, in the presence of an electrically conductive solution such as tissue culture medium. Adhering cells disrupt the interaction between the electrodes and the solution and thus impede electron flow. The impedance of electron flow caused by adherent cells is calculated via computed mathematical algorithms and reported as an arbitrary parameter called Cell Index (CI), the magnitude of which reflects the rate and extent of cell proliferation. Briefly, patient-derived dermal fibroblasts and control hNDFs were cultured in fibroblast growth medium (ATCC) and seeded at a density of 2500 cells per well in a final volume of 100 µl into a 96-well RTCA E-plate. The E-plate was placed in the RTCA SP station housed inside a standard CO2 cell culture incubator. Cellular impedance data (CI value) was continuously recorded over a 4-day period using the xCELLigence RTCA Software installed on the control unit outside the incubator. Subsequently CI curves were analyzed and the Slope parameters were plotted to determine cell proliferation activity over time. Data were presented as average +SEM. A two-tailed Student's *t*-test was used to calculate statistical significance.

Migration Assay: An xCELLigence RTCA Double Purpose (DP) instrument (Agilent) was used to monitor real-time cell migration. This instrument utilizes an electronically integrated Boyden chamber, the CIM plate, to make continuous measurements of electrical impedance generated by cell migration through the microporous membrane of the upper chamber to the gold microelectrodes-fused bottom surface of the lower chamber. Briefly, patient-derived dermal fibroblasts and control hNDFs, or Alms1 siRNA treated neonatal rat cardiac fibroblasts (NRCFs) and scramble treated NRCFs, or ALMS1 siRNA treated hNDFs and scramble treated hNDFs, were cultured in DMEM with 10% FBS and passaged the day prior to the experiment. Cells in the range of 60-80% confluence were trypsinized using 0.05%/Trypsin/EDTA solution, washed once with serum-free medium (SFM), and resuspended in SFM. Subsequently cells were seeded at a density of 10,000/20,000 cells per well in a final volume of 100 µl into the top chamber of a CIM-Plate16 (10,000 cells per well for human dermal fibroblasts; 20,000 cells per well for NRCFs). The CIM plate containing the cells were placed at room temperature for 30 minutes to allow the cells to settle down to the bottom surface (the microporous membrane) of the upper chamber, and then loaded into the DP System inside the incubator. Impedance signal was measured with an interval of 15 minutes for 24 hours. Subsequently CI curves were analyzed and the Slope parameters were plotted to determine cell migration activity. Data were presented as average +SEM. A two-tailed Student's t-test was used to calculate statistical significance.

FACS Analysis: pEFE patient-derived dermal fibroblasts and control hNDFs were cultured in DMEM with 10% FBS to 40%-50% confluence, followed by cell cycle synchronization in serum-free DMEM for 16 hours. The cells were subsequently treated with 10μM Edu (5-ethennyl-2-deoxyuridine) in DMEM with 10% FBS for 24 hours, incubated in DMEM with 10%FBS for another 24 hours, followed by fixation with 4% paraformaldehyde (PFA). The cells were then subjected to FACS analysis.

SUPPLEMENTAL REFERENCES

1. Touma M, Kang X, Gao F, Zhao Y, Cass AA, Biniwale R, et al. Wnt11 regulates cardiac chamber development and disease during perinatal maturation. JCI Insight. 2017;2:e94904.
2. Touma M, Kang X, Zhao Y, Cass AA, Gao F, Biniwale R, et al. Decoding the Long Noncoding RNA During Cardiac Maturation: A Roadmap for Functional Discovery. Circ Cardiovasc Genet. 2016;9:395-407.
3. McKenna A, Hanna M, Bank E, Sivachenko A, Cibulskis K, Kernytsky A, Garimella K, Altshuler D, Gabriel S, Daly M, DePristo MA. The Genome Analysis Toolkit: A MapReduce framework for analyzing next-generation DNA sequencing data. Genome Res. 2010;20(9):1297-303.

SUPPLEMENTAL FIGURES LEGENDS

Supplemental Figure 1. Bioinformatics Algorithms for Whole Exome Sequencing (WES) and RNA-seq

Supplemental Figure 2. FACS analysis of proliferation activity in pEFE fibroblasts compared to hNDFs. Despite enhanced Edu incorporation, no difference was identified in the mitotic phase (G2/M) phase.

Supplemental Figure 3. ALMS1 Suppression in Neonatal Dermal Fibroblasts (hNDFs) Does Not Affect Fibroblast proliferation.

A. Proliferation assay of ALMS1 siRNA-treated vs. scramble treated hNDFs showing no deference in proliferation activity. The proliferation assay was performed using an xCELLigence RTCA SP instrument over an 85-hour period. 2500 cells per well were seeded into the 96-well RTCA E-plate. N=6 biological replicates per group.

B. Quantitative analysis of fibroblast proliferation assay shown in B.

SUPPLEMENTAL TABLES LEGENDS

Supplemental Table 1 (Attached Excel Sheet 1): Primary gene list of known cardiomyopathy genes.

Supplemental Table 2 (Attached Excel Sheet 2): Runs of homozygosity (exceeding 5 MBs) identified in the pEFE family suggest that both parents descend from a genetically-related community.

Supplemental Table 3 (Attached Excel Sheet 3): Lists of top significant differentially expressed genes (DGEs) (pEFE fibroblasts_vs_hNDFs) enriched in cell adhesion molecules functional ontology terms. Red: Upregulated, Green: Downregulated.

Supplemental Table 4 (Attached Excel Sheet 4): Lists of top significant differentially expressed genes (DGEs) (pEFE fibroblasts_vs_hNDFs) enriched in TGFβ/BMP signaling pathway. Red: Upregulated, Green: Downregulated.

Supplemental Table 5 (Attached Excel Sheet 5). Lists of top significant differentially expressed genes (DGEs) enriched in extracellular matrix (ECM) functional terms.

Supplemental Table 6 (Attached Excel Sheet 6): Lists of top significant differentially expressed genes (DGEs) enriched in retinol, insulin, and leptin signaling pathways.
