# Supplementary material for: Recessive ciliopathy mutations in primary endocardial fibroelastosis: a rare neonatal cardiomyopathy in a case of Alstrom syndrome
Source: J Mol Med (Berl). 2021 Aug 13;99(11):1623–38. doi: 10.1007/s00109-021-02112-z (PMC8541947; doi:10.1007/s00109-021-02112-z)
Supplement: Supplementary file 3 — Supplementary file3 (PDF 226 KB) [file 109_2021_2112_MOESM3_ESM.pdf]

Supplemental Materials

TITLE

Integrated Genomics Analysis Identifies Recessive Ciliopathy Mutations in Primary Endocardial Fibroelastosis: a Rare Neonatal Cardiomyopathy

AUTHORS AND AFFILIATIONS

Yan Zhao[1-3], Lee-kai Wang[4], Ascia Eskin[5], Xuedong Kang[1-3], Viviana M. Fajardo[1], Zubin Mehta[1-3], Stacy Pineles[6], Ryan J. Schmidt[7], Aaron Nagiel[8,9], the UCLA Congenital Heart Defects BioCore Faculty[$], Gary Satou[1], Meena Garg[1], Myke Federman[1], Leigh C. Reardon[1,10], Steven L. Lee[1], Reshma Biniwale[1,11], Wayne W. Grody[1,12], Nancy Halnon[1], Negar Khanlou[12], Fabiola Quintero-Rivera[13], Juan Alejos[1], Gregory Fishbein[12], Glen Van Arsdell[1,11], Stanley F. Nelson[1,4,5], Marlin Touma[1-3,14,15].

1. Department of Pediatrics, David Geffen School of Medicine, University of California Los Angeles, Los Angeles, CA.
2. Neonatal/Congenital Heart Laboratory, Cardiovascular Research Laboratory, University of California Los Angeles, Los Angeles, CA.
3. Children's Discovery and Innovation Institute, Department of Pediatrics, David Geffen School of Medicine, University of California Los Angeles, Los Angeles, CA.
4. Institute for Precision Health, David Geffen School of Medicine, University of California Los Angeles, Los Angeles, CA.
5. Department of Human Genetics, David Geffen School of Medicine, University of California Los Angeles, Los Angeles, CA.
6. Department of Ophthalmology, David Geffen School of Medicine, University of California Los Angeles, Los Angeles, CA.
7. Department of Pathology and Laboratory Medicine, Children's Hospital Los Angeles, Los Angeles, CA.
8. The Vision Center, Department of Surgery, Children's Hospital Los Angeles, Los Angeles, CA.
9. Roski Eye Institute, Department of Ophthalmology, University of Southern California. Los Angeles, CA.
10. Ahmanson/UCLA Adult Congenital Heart Disease Center. Department of Medicine, David Geffen School of Medicine, University of California Los Angeles,
Los Angeles, CA.
11. Department of Cardiothoracic Surgery, David Geffen School of Medicine, University of California Los Angeles, Los Angeles, CA.
12. Department of Pathology and Laboratory Medicine, David Geffen School of Medicine, University of California Los Angeles, Los Angeles, CA.
13. Department of Pathology and Laboratory Medicine, School of Medicine, University of California Irvine, Irvine, CA.
14. The Molecular Biology Institute, David Geffen School of Medicine, University of California Los Angeles, Los Angeles, CA.
15. Eli and Edythe Broad Stem Cell Research Center, David Geffen School of Medicine, University of California Los Angeles, Los Angeles, CA.

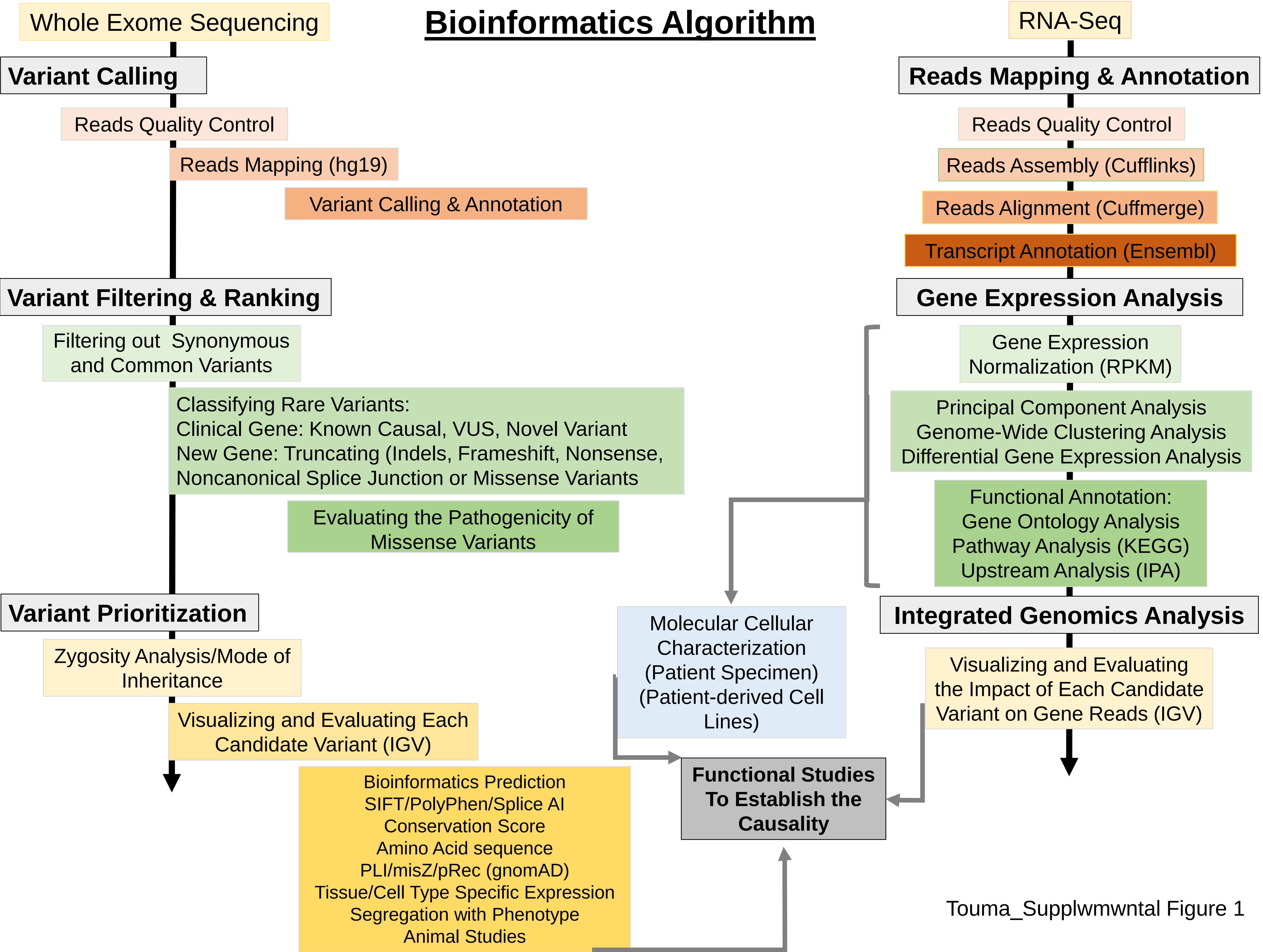

Touma_Supplwmwntal Figure 1

Supplemental Figure 1. Bioinformatics Algorithms for Whole Exome Sequencing (WES) and RNA-seq.

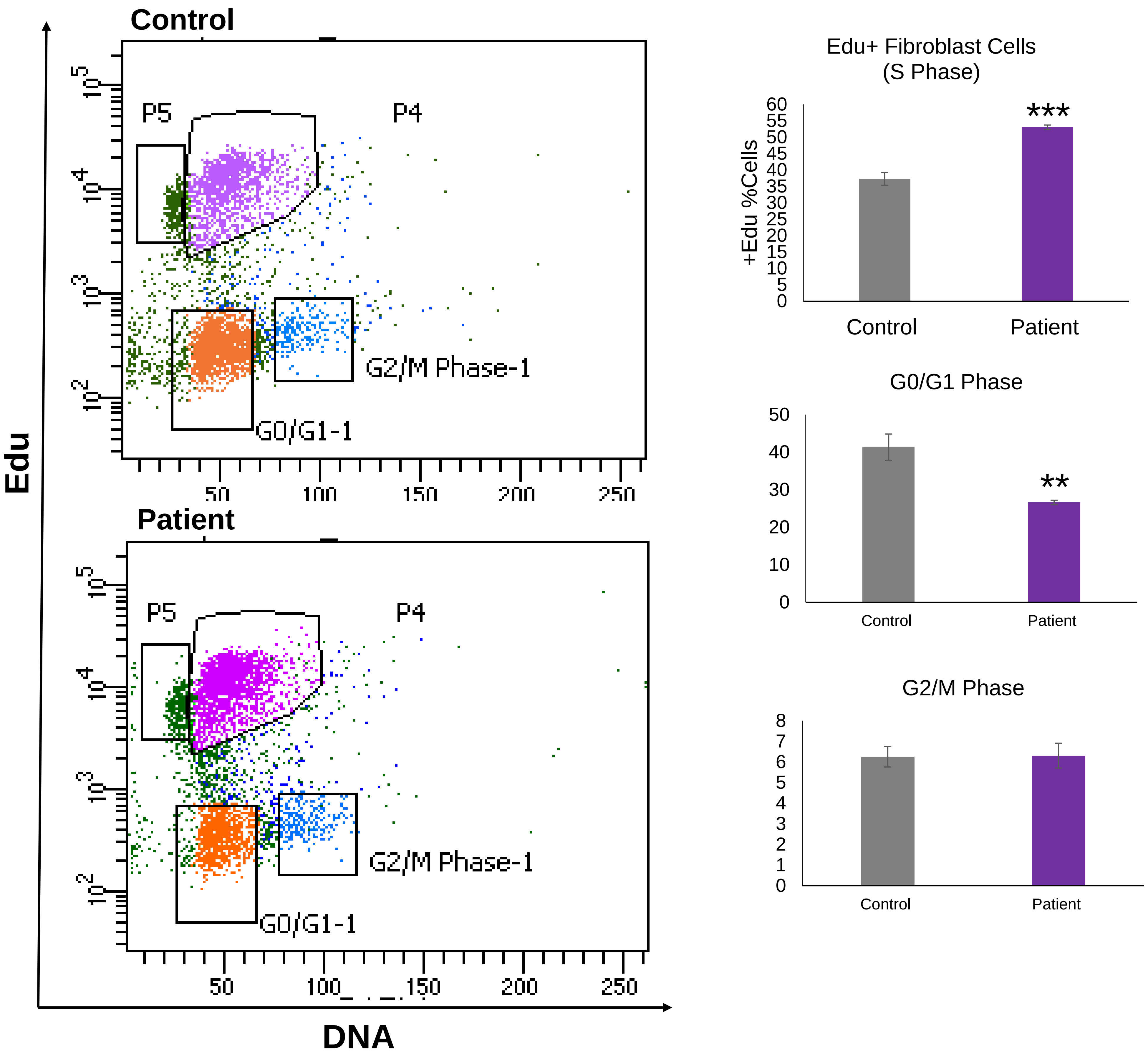

Supplemental Figure 2. FACS Analysis of proliferation activity of pEFE4 fibroblasts compared to hNDFs. Despite enhanced Edu incorporation, no difference was identified in the mitotic phase (G2/M).

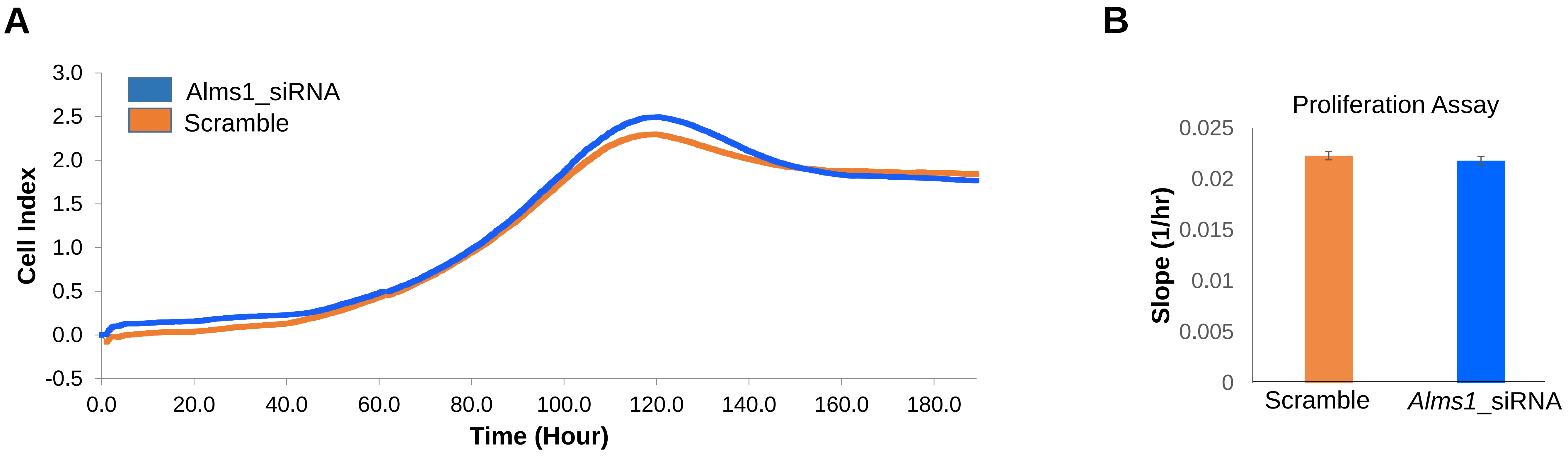

Supplemental Figure 3. ALMS1 Suppression in Neonatal Dermal Fibroblasts (hNDFs) Enhances Migration Activity.

A. Proliferation assay of ALMS1 siRNA-treated vs. scramble treated hNDFs showing no deference in proliferation activity. The proliferation assay was performed using an xCELLigence RTCA SP instrument over an 85-hour period. 2500 cells per well were seeded into the 96-well RTCA E-plate. N=6 biological replicates per group.
B. Quantitative analysis of fibroblast proliferation assay shown in B.

**Supplemental Table 1.** Cardiomyopathy Gene List		
ABCC9	*JUP*	*PSEN2*
ACTC1	*LDB3*	*RBM20*
ACTN2	*LMNA*	*RYR2*
BAG3	*MYBPC3*	*SCN5A*
CASQ2	*MYH6*	*SGCD*
CSRP3	*MYH7*	*TAZ*
DES	*MYL2*	*TCAP*
DMD	*MYL3*	*TMPO*
DNAJC19	*MYLK2*	*TNNC1*
DSG2	*MYOZ2*	*TNNI3*
DSP	*NEXN*	*TNNT2*
DTNA	*PKP2*	*TPM1*
EYA4	*PLN*	*TTN*
FCMD	*PRKAG2*	*VCL*
FKTN	*PSEN1*	

Supplemental Table 1 (Attached Excel Sheet 1). Primary gene list of known cardiomyopathy genes

Supplemental Table 2 (Attached Excel Sheet 2). Runs of Homozygosity Identified in pEFE Proband Family.

Proband

FID	IID	PHE	CHR	SNP1	SNP2	POS1	POS2	KB
1	1	2	1	rs5702	rs7417104	71331430	78312788	6981.358
1	1	2	1	rs302792	rs9651170	99631074	104768887	5137.813
1	1	2	2	rs2542529	rs10185195	71615571	78742826	7127.255
1	1	2	2	rs4852584	rs10182351	80793411	85872285	5078.874
1	1	2	6	rs9272535	rs742493	32606756	40998167	8391.411
1	1	2	14	rs17113841	rs8011060	28787738	35546373	6758.635
1	1	2	17	rs2280269	rs10852789	75207966	81006286	5798.32
1	1	2	21	rs12626187	rs2834172	28083522	34655623	6572.101

Mother

FID	IID	PHE	CHR	SNP1	SNP2	POS1	POS2	KB
1	1	2	2	rs11887088	rs1568540	114821532	120362407	5540.875
1	1	2	4	rs7690043	rs11131619	57116323	66658551	9542.228
1	1	2	20	rs293565	rs6016352	31097860	39053491	7955.631

Father

FID	IID	PHE	CHR	SNP1	SNP2	POS1	POS2	KB
1	1	2	3	rs13085937	rs7644510	182178216	190980797	8802.581
1	1	2	4	rs9307678	rs2305934	81308107	89744242	8436.135

Cell adhesion molecules (Cams)

Symbol	Description	LogFC_ pEFE_vs_hNDFs
BMPR1B	bone morphogenetic protein receptor type 1B	9.074819
HLA-C	major histocompatibility complex, class I, C	8.794926
HLA-B	major histocompatibility complex, class I, B	7.731426
CNTN1	contactin 1	6.648164
HLA-E	major histocompatibility complex, class I, E	6.082751
XG	Xg blood group	5.390579
MFAP3L	microfibril associated protein 3 like	4.717871
NRXN2	neurexin 2	4.606978
NFASC	neurofascin	4.040345
NCAM1	neural cell adhesion molecule 1	4.032204
LINGO1	leucine rich repeat and Ig domain containing 1	3.764783
CD4	CD4 molecule	3.638799
JAM2	junctional adhesion molecule 2	3.155378
HLA-A	major histocompatibility complex, class I, A	2.802985
INHBB	inhibin beta B subunit	2.77062
CHRD	chordin	2.658392
SDC2	syndecan 2	2.579577
BMP6	bone morphogenetic protein 6	2.518243
ITGB8	integrin subunit beta 8	2.492423
NTNG1	netrin G1	2.429109
ACVR1C	activin A receptor type 1C	2.412751
NEO1	neogenin 1	2.361982
VCAN	versican	2.315403
ID2	inhibitor of DNA binding 2	2.146962
ITGA9	integrin subunit alpha 9	2.023498
CD40	CD40 molecule	-2.00894
FST	follistatin	-2.13182
NLGN4X	neuroligin 4, X-linked	-2.26282
ICOSLG	inducible T cell costimulator ligand	-2.26965
PTPRF	protein tyrosine phosphatase, receptor type F	-2.59948
L1CAM	L1 cell adhesion molecule	-4.47879
GDF6	growth differentiation factor 6	-4.51857
BMP4	bone morphogenetic protein 4	-4.91715
KCP	kielin/chordin-like protein	-5.36249
ESAM	endothelial cell adhesion molecule	-5.36683
IRX4	iroquois homeobox 4	-5.83312
CNTNAP3B	contactin associated protein like 3B	-9.21321
NLGN4Y	neuroligin 4, Y-linked	-11.4955

Touma_Supplemental Table 3

TGFB/BMP Signaling

Symbol	Description	LogFC_ pEFE_vs_hNDFs
BMPR1B	bone morphogenetic protein receptor type 1B	9.074819
ID4	inhibitor of DNA binding 4, HLH protein	3.728158
INHBB	inhibin beta B subunit	2.77062
CHRD	chordin	2.658392
BMP6	bone morphogenetic protein 6	2.518243
ACVR1C	activin A receptor type 1C	2.412751
ID2	inhibitor of DNA binding 2	2.146962
FST	follistatin	-2.13182
GDF6	growth differentiation factor 6	-4.51857
BMP4	bone morphogenetic protein 4	-4.91715
KCP	kielin/chordin-like protein	-5.36249
IRX4	iroquois homeobox 4	-5.83312

Touma_Supplemental Table 4

ECM Proteins

Symbol	Desccription	logFC_ pEFE4_vs_hNDFs
COL25A1	collagen type XXV alpha 1 chain	4.449568444
COL25A1	collagen type XXV alpha 1 chain	4.449568444
COL5A3	collagen type V alpha 3 chain	1.667328567
COL24A1	collagen type XXIV alpha 1 chain	1.440059616
COL24A1	collagen type XXIV alpha 1 chain	1.440059616
COL28A1	collagen type XXVIII alpha 1 chain	1.258511414
COL17A1	collagen type XVII alpha 1 chain	1.07237257
COL6A3	collagen type VI alpha 3 chain	1.0409639
COL6A1	collagen type VI alpha 1 chain	0.851871098
COL8A1	collagen type VIII alpha 1 chain	0.489400689
COL8A2	collagen type VIII alpha 2 chain	0.245970073
COL4A1	collagen type IV alpha 1 chain	0.173494283
COL1A2	collagen type I alpha 2 chain	0.133069935
COLQ	collagen like tail subunit of asymmetric acetylcholinesterase	0.120499188
COL4A2	collagen type IV alpha 2 chain	0.035246212
PCOLCE	procollagen C-endopeptidase enhancer	0.020808128
COLGALT2	collagen beta(1-O)galactosyltransferase 2	-0.071101654
COL5A1	collagen type V alpha 1 chain	-0.080511415
LOC101928841	collagen alpha-1(II) chain-like	-0.152298845
PLOD1	procollagen-lysine,2-oxoglutarate 5-dioxygenase 1	-0.173134475
COL1A1	collagen type I alpha 1 chain	-0.25875761
COL5A2	collagen type V alpha 2 chain	-0.278953002
PLOD2	procollagen-lysine,2-oxoglutarate 5-dioxygenase 2	-0.454298159
CCBE1	collagen and calcium binding EGF domains 1	-0.575508234
COL14A1	collagen type XIV alpha 1 chain	-0.644394935
COL11A1	collagen type XI alpha 1 chain	-0.709108586
PCOTH	Pro-X-Gly collagen triple helix like repeat containing	-0.782708012
COL18A1	collagen type XVIII alpha 1 chain	-1.108517863
COL4A5	collagen type IV alpha 5 chain	-1.169761524
COL12A1	collagen type XII alpha 1 chain	-1.329394079
COL7A1	collagen type VII alpha 1 chain	-1.417603488
COL4A6	collagen type IV alpha 6 chain	-1.719620938
COL27A1	collagen type XXVII alpha 1 chain	-1.814119599
COL13A1	collagen type XIII alpha 1 chain	-2.460601079
COL21A1	collagen type XXI alpha 1 chain	-2.515387356
COL15A1	collagen type XV alpha 1 chain	-3.403323249

Touma_ Supplemental Table 5

Retinol signaling

Symbol	Description	logFC_ pEFE4_vs_hNDFs
LHX2	LIM homeobox 2	6.743271
RBP1	retinol binding protein 1	6.156071
RARB	retinoic acid receptor beta	4.381661
BRINP2	BMP/retinoic acid inducible neural specific 2	3.379813
BRINP1	BMP/retinoic acid inducible neural specific 1	2.744107
SCT	secretin	2.526169
STRA6	stimulated by retinoic acid 6	-2.43267
ISL2	ISL LIM homeobox 2	-2.82875
RPE65	RPE65, retinoid isomerohydrolase	-3.2927
PDE6B	phosphodiesterase 6B	-3.48619
GRHL1	grainyhead like transcription factor 1	-5.20243
RARRES2	retinoic acid receptor responder 2	-5.43528
ISL1	ISL LIM homeobox 1	-7.70082

Insulin signaling

Symbol	Desccription	logFC_ pEFE4_vs_hNDFs
IGFBP1	insulin like growth factor binding protein 1	2.491068
IGFBP5	insulin like growth factor binding protein 5	1.19158
IGFBP3	insulin like growth factor binding protein 3	1.053346
IGFBP2	insulin like growth factor binding protein 2	0.724424
PIGF	phosphatidylinositol glycan anchor biosynthesis class F	0.441855
IGFLR1	IGF like family receptor 1	0.303328
IGFBP7	insulin like growth factor binding protein 7	0.078395
IGF1R	insulin like growth factor 1 receptor	-0.04358
IGF2BP2	insulin like growth factor 2 mRNA binding protein 2	-0.15814
IGF2BP1	insulin like growth factor 2 mRNA binding protein 1	-0.23667
IGFBP6	insulin like growth factor binding protein 6	-0.37664
IGF2R	insulin like growth factor 2 receptor	-0.51018
IGF2BP3	insulin like growth factor 2 mRNA binding protein 3	-0.56958
IGFBP4	insulin like growth factor binding protein 4	-0.61558
IGF2	insulin like growth factor 2	-1.97944

Leptin Signaling

Symbol	Description	LogFC_ pEFE4_vs_hNDFs
MCHR1	melanin concentrating hormone receptor 1	4.411475969
CUX1	cut like homeobox 1	0.261378559
AGRP	agouti related neuropeptide	-1.13341199
MYO5B	myosin VB	-1.222706292
LEPR	leptin receptor	-1.66313673
SIM1	single-minded family bHLH transcription factor 1	-1.680886035
LEPR	leptin receptor	-1.66313673

Touma_Supplemental Table 6
